# Determinants of demand for total hip and knee arthroplasty: a systematic literature review

**DOI:** 10.1186/1472-6963-12-225

**Published:** 2012-07-30

**Authors:** Rubén E Mújica Mota, Rosanna Tarricone, Oriana Ciani, John FP Bridges, Mike Drummond

**Affiliations:** 1 Institute for Health Services Research, University of Exeter, Veysey Building, Salmon Pool Lane, Exeter, EX2 4SG, , UK; 2 Centre for Research in Healthcare Management, Universita Bocconi, Via Roentgen 1, 20136, Milan, Italy; 3 Johns Hopkins Bloomberg School of Public Health, 624 N. Broadway, Room 689, Baltimore, MD, 21205, USA; 4 Centre for Health Economics, Alcuin 'A' Block, University of York, Heslington, York, YO10 5DD, UK

**Keywords:** Orthopaedic implant, Arthroplasty, Hip, Knee, Demand, Need, Equity, Preferences, Patient selection, Osteoarthritis, Decision to operate, Socio-economic disparities, Total joint replacement

## Abstract

**Background:**

Documented age, gender, race and socio-economic disparities in total joint arthroplasty (TJA), suggest that those who need the surgery may not receive it, and present a challenge to explain the causes of unmet need. It is not clear whether doctors limit treatment opportunities to patients, nor is it known the effect that patient beliefs and expectations about the operation, including their paid work status and retirement plans, have on the decision to undergo TJA. Identifying socio-economic and other determinants of demand would inform the design of effective and efficient health policy. This review was conducted to identify the factors that lead patients in need to undergo TJA.

**Methods:**

An electronic search of the Embase and Medline (Ovid) bibliographic databases conducted in September 2011 identified studies in the English language that reported on factors driving patients in need of hip or knee replacement to undergo surgery. The review included reports of elective surgery rates in eligible patients or, controlling for disease severity, in general subjects, and stated clinical experts’ and patients’ opinions on suitability for or willingness to undergo TJA. Quantitative and qualitative studies were reviewed, but quantitative studies involving fewer than 20 subjects were excluded. The quality of individual studies was assessed on the basis of study design (i.e., prospective versus retrospective), reporting of attrition, adjustment for and report of confounding effects, and reported measures of need (self-reported versus doctor-assessed). Reported estimates of effect on the probability of surgery from analyses adjusting for confounders were summarised in narrative form and synthesised in odds ratio (OR) forest plots for individual determinants.

**Results:**

The review included 26 quantitative studies−23 on individuals’ decisions or views on having the operation and three about health professionals’ opinions-and 10 qualitative studies. Ethnic and racial disparities in TJA use are associated with socio-economic access factors and expectations about the process and outcomes of surgery. In the United States, health insurance coverage affects demand, including that from the Medicare population, for whom having supplemental Medicaid coverage increases the likelihood of undergoing TJA. Patients with post-secondary education are more likely to demand hip or knee surgery than those without it (range of OR 0.87-2.38). Women are as willing to undergo surgery as men, but they are less likely to be offered surgery by specialists than men with the same need. There is considerable variation in patient demand with age, with distinct patterns for hip and knee. Paid employment appears to increase the chances of undergoing surgery, but no study was found that investigated the relationship between retirement plans and demand for TJA. There is evidence of substantial geographical variation in access to joint replacement within the territory covered by a public national health system, which is unlikely to be explained by differences in preference or unmeasured need alone. The literature tends to focus on associations, rather than testing of causal relationships, and is insufficient to assess the relative importance of determinants.

**Conclusions:**

Patients’ use of hip and knee replacement is a function of their socio-economic circumstances, which reinforce disparities by gender and race originating in the doctor-patient interaction. Willingness to undergo surgery declines steeply after the age of retirement, at the time some eligible patients may lower their expectations of health status achievement. There is some evidence that paid employment independently increases the likelihood of operation. The relative contribution of variations in surgical decision making to differential access across regions within countries deserves further research that controls for clinical need and patient lifestyle preferences, including retirement decisions. Evidence on this question will become increasingly relevant for service planning and policy design in societies with ageing populations.

## Background

Total hip arthroplasty (THA) and total knee arthroplasty (TKA) are effective for reducing pain and restoring the function and mobility of patients with severe arthritis [1]. In the United States from 1993 through 2005, TKA operations increased almost 2.5 times (from 200,216 to 497,419), while THA increased 1.7 fold (from 135,992 to 237,645). During the same period, the ratio of revisions to primary replacements decreased from 20% to 15.7% for THA, and from 9.2% to 8% for TKA [2]. In the UK, use of TKA rose, while THA remained stable over the period [3].

Changes in utilisation reflect an increasing need for surgery as a treatment for osteoarthritis (OA) derived from longer life expectancies, a rising elderly population, and an increased prevalence of obesity, which may also explain the faster increase of TKA relative to THA [4]. Improvements in devices, and in surgical and anaesthetic techniques, have widened the age range of patients eligible to receive these procedures [2,3].

These developments have taken place alongside observed utilisation differences by age, gender, and socio-economic status [5]. Such disparities warrant further research to establish the extent to which they reflect variations in disease risk or inequities in healthcare services [5,6]. In particular, women and individuals aged 70–80 are in greater need and have experienced larger increases in utilisation in recent years [3], but they face greater barriers to treatment access. Geographical barriers may also exist, as evidenced by the low rates of utilisation of deprived residential areas served by public healthcare systems [6] and variations in utilisation across areas of the United States [7,8].

According to their clinical training and field of medical specialty, health professionals may have differing views about whether a given person is a candidate for surgery [9,10]. Nevertheless, there is general agreement on some indications. For TKA, these include pain not controlled by medication and functional limitations, such as inability to walk at least one block [11]. As for contraindications, orthopaedists [12,13] and orthopaedic surgeons [14] cite some that include dementia and major psychiatric disorders, rheumatologists and orthopaedic surgeons name peripheral vascular disease [11,14], and surgeons and orthopaedists refer to alcohol or drug abuse [4,12]. In addition, orthopaedic surgeons tend to agree that those younger than 55 and those with a physically demanding job are less likely candidates for TKA [14]. Less common contraindications for TKA include severe hip OA, quadriceps lag/weak quadriceps, obesity, and knee sepsis for more than a year [15].

Many patients who might otherwise benefit do not undergo surgery as a matter of preference [16,17]. It is critical to determine whether the decision to undergo surgery is systematically related to the underlying health of patients or to characteristics such as their socio-economic status, which might determine their ability to benefit from and access surgery. Identifying the determinants of demand by those in need of surgery may inform planning for future orthopaedic resource needs, the design of interventions to address health inequities, and efficient targeting of resources.

The purpose of this paper is to report a systematic literature review that was conducted to identify the factors that lead patients in need to undergo THA or TKA. The review considered studies including patients who may be deemed eligible for elective surgery due to self-reported chronic pain, measured severity of disease, or doctor assessment, in order to ascertain the effect that clinical, demographic and socio-economic patient characteristics had on their probability of undergoing surgery or being recommended for surgery by doctors. Prospective and retrospective longitudinal and cross-sectional studies were considered for review.

## Methods

An electronic search was undertaken in Medline (Ovid) and Embase up to 6 September 2011, using terms related to ‘need’, ‘decision to undergo surgery’, and ‘hip’ or ‘knee’ replacement (see Additional file 1). The titles and abstracts were screened to identify studies of predictors of doctors’ referral or recommendation for surgery, and patients’ decisions to undergo surgery.

Studies were eligible for full-text review if they measured therapy utilisation while adjusting for need. The full text of studies reporting rates or likelihood of elective operations or measuring need or severity of illness were retrieved to ascertain whether rates of TJA receipt, offer or acceptance specific to individuals who might be eligible for surgery (on the basis of diagnosis of musculoskeletal condition, chronic pain, or functional limitations, or indicated as such by a health professional) had been reported and should therefore be considered for review. Studies that only presented rates on a per capita basis for the general population were excluded.

Studies of doctors’ opinions about clinical characteristics or signs and symptoms in hypothetical patients were excluded unless they were validated against outcomes in actual patients. This restriction had the effect of excluding the literature on clinical and radiological opinion and health professionals’ attitudes to risk and preference that, while relevant, would have added the complexity of reconciling stated with actual professional practice.

Exclusions also applied to reports of prevalence of need or of patients opinions’ on surgery that had no subsequent analysis of predictors of incidence of operation or willingness to undergo surgery; trends in total number of hip or knee operations by providers or regions without account of patient need; preference elicitation studies in the general public; probability of surgery in any joints (combined with shoulder or other joints); healthcare consumption (e.g. costs) or indication (elective and non-elective) without specific reports for elective hip or knee surgery; and patient-doctor interaction studies without reference to a referral or operation decision. Quantitative studies with fewer than 20 patients were also excluded. References in the identified studies, were hand searched for additional relevant studies, which were screened according to the same process as applied to studies electronically searched.

A standardised form was used to extract information from included studies on study dates, place, setting, design and follow-up; objectives and sample characteristics; measured determinants associated with patients, doctors, setting or place; methods to adjust for confounding; and estimated independent effects of determinants (see Additional files 2, 3, 4, 5). Included studies were evaluated for the regional or national representativeness of their patient or clinician samples, and four attributes of internally valid observational analyses of demand determinants: prospective/longitudinal study and analysis; adjustment for socio-economic, clinical, and demographic confounders for estimating effects of determinants; use of both patient and doctor-assessed measures of need; and the reporting of attrition. For quantitative studies, each of these four elements of internal validity was rated as 1 if the requirement was met and 0 otherwise, and the overall sum across elements represented the quality score. Qualitative studies were not scored but were used to complement the information from quantitative studies. Screening of titles and abstracts, data extraction, and quality scoring were performed independently by two reviewers, who resolved their differences by discussion. The study protocol is available from the authors upon request (see Additional file 2 for PRISMA statement).

Given the heterogeneity of study designs and reporting practice in the literature, results from different studies were not synthesized quantitatively, but were presented in forest plots that made it possible to compare results across similar independent reports. The odds ratio (OR) and associated confidence interval (CI), the most commonly reported measure of effect, are presented for demand predictors. The review considered only ORs derived from (logistic) regression analyses that controlled for multiple covariates simultaneously. The major relative strengths or weaknesses of studies are noted, primarily in relation to the extent to which causal relationships may be inferred, and confounding discounted, from their results.

## Results

**Figure 1:**
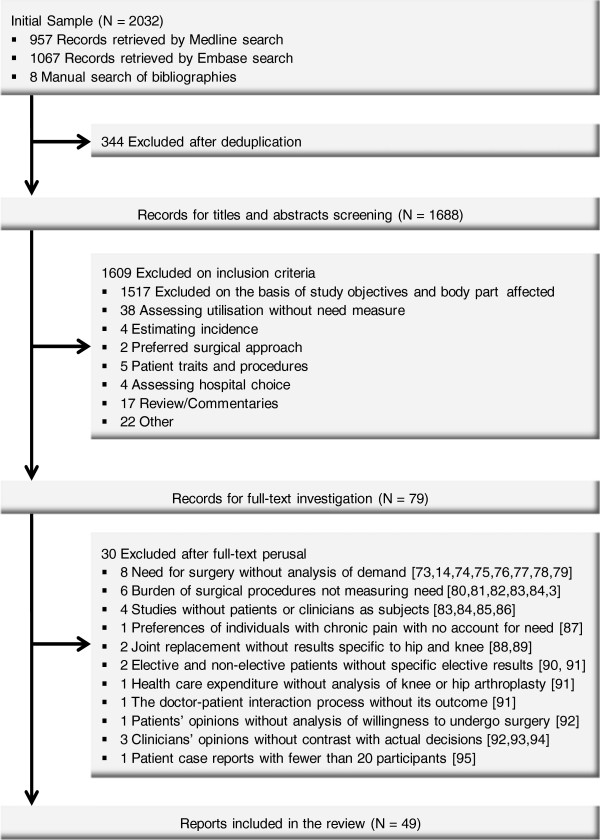
Process of identification of demand studies.

The search yielded 2024 hits, for 1680 different records (923 in Medline and 757 in Embase). After the elimination of studies judged to be irrelevant because of their patient population (e.g., involving problems in joints other than hip or knee, sickle cell disease, osteonecrosis or malignancies), focus (e.g., health outcomes assessment, patient management practice alongside TKA or THA), scope (e.g., utilisation rates without accounting for patient need) or publication type (i.e., reviews and commentaries), 48 studies from Medline and an additional 23 studies from Embase remained (see Figure 1). Retrieval of full articles led to an additional 28 exclusions. A manual search through the bibliographies of the retrieved publications identified eight additional publications, six of which were included. In total, 49 publications were included in the review. Thirteen of these were extensions of initial studies and were thus reviewed together with the seminal study, constituting evidence on 36 distinct studies.

The studies were conducted in Australia, Canada (4), Denmark, France, Japan, The Netherlands, New Zealand, Spain (2), the UK (10), and the United States (14). Among these, 30 involved patients as subjects, whilst 6 studies included doctors. Three of the studies with doctors reported priority or referral algorithms.

In the studies on patients, need for total joint arthroplasty (TJA) was sometimes measured using standardised methods such as the WOMAC disease-specific tool (5 studies), the Lequesne index (3), or the New Zealand score (3). In other studies, need was established through a self-reported condition or doctor diagnosis of arthritis, rheumatism, or arthritis-related visit to a doctor (3), diagnosis by a specialist physician (8), or chronic pain complaints or referrals to a specialist (7). The remaining study implicitly controlled for need by extrapolation using regression methods. Among the studies on patients, 7 were qualitative, 5 were quantitative assessments of patient views on surgery, 2 were concerned with willingness to change surgeons to reduce waiting time, and the remaining 16 were quantitative studies of factors behind receipt of surgery (see Additional file 3). Most of the studies included a mixed sample of retired and working-age individuals; only 4 of the quantitative studies involved mostly or exclusively patients older than 65.

The quality scoring of quantitative studies (excluding the three studies on referral algorithms) is presented in Table 1. The overall scores are summarised in Table 2. Two studies, one related to doctors’ decision making and one involving patients as subjects, met all the quality criteria for validity and were also potentially applicable to general practice. Three studies met only one out of four validity criteria. The majority of studies reported the effect of demographic characteristics on receipt of surgical treatment or willingness to consider or pay for surgery. Only 13 out of the 26 studies accounted for need based on a clinical evaluation or radiograph. Seven studies reported the effect of education, five studies that of health insurance, four that of income, and two reported on all three factors. The results are described in the next section, first for studies involving patients as subjects and then for those involving doctors.

**Table 1:** Quality checklist

**QUESTION**	**1**	**2**	**3**	**4**	**5**	**6**	**7**	**8**	**9**	**10**	**11**	**12**	**13**	**14**	**15**	**16**	**17**	**18**	**19**	**20**	**21**	**22**	**23**	**24**	**25**	**26**
**Is the sample representative of patients and practice in region/country?**		■		■	■			■		■	■	■		■	■	■	■	■	■	■			■	■		■
**Was the study prospective?**		■	■	■		■		■	■	■	■	■			■		■	■	■	■	■	■	■	■	■	■
**Did study report the country/year of study?**	■	■	■	■	■	■	■	■	■	■	■	■	■	■	■	■	■	■	■	■		■	■	■	■	■
**Did it report the effects of patient characteristics / factors?**																										
- **Age**				■		■	■	■	■	■	■	■			■	■	■		■	■			■	■	■	■
- **Gender**	■		■	■		■	■	■	■	■	■	■			■	■				■			■	■		■
- **Race**					■				■	■			■		■							■	■	■		
- **BMI**				■					■	■		■					■						■			■
- **Marital status**		■		■					■														■	■		
- **Severity of disease**				■		■		■	■	■	■	■		■		■	■		■	■			■			
- **Bilateral/Unilateral**																										■
- **Work status**				■			■			■		■											■			
- **Income**									■	■	■	■										■				
- **Health insurance**					■		■	■	■	■																
- **Education**					■	■			■	■	■	■										■	■	■		
- **Location of residence**				■								■			■											
- **Supply of surgeons**															■											
- **Other**	■			■		■	■	■	■	■	■	■	■		■		■	■	■	■			■	■		■
**Did study report attrition?**	■		■	■	■	■	■			■	■	■			■	■	■	■		■		■	■	■	■	
**Did it use a patient reported outcome tool to define need?**		■		■		■	■		■	■	■	■			■	■	■		■	■	■		■	■	■	■
**If so, did study report:**																										
- **Functioning**		■		■			■		■	■	■	■		■	■	■	■	■		■	■			■	■	■
- **Mobility**		■		■			■				■	■		■	■	■	■	■		■	■		■	■	■	
- **Pain**		■		■			■				■	■		■	■	■	■	■	■	■	■		■	■	■	
**Did the study account for doctor assessed need?**		■	■	■					■			■		■			■		■	■	■					■

1. Birk and Henriksen, 2006 [54] 2. Birrel et al., 2003 [22] 3. Borkhoff et al., 2008 [55] 4. Boutron et al., 2008 [60] 5. Card et al., 2008 [47] 7. Cross et al., 2000 [79] 13. Ibrahim et al., 2002 [39] 19. Momohara et al., 2007 [23] 26. Yong., et al. 2004 [48].

6. Conner-Spady et al., 2008 [53] 7. Cross et al., 2000 [79] 8. Dunlop et al., 2003 [29] 9. George et al., 2008 [20] 10. Hanchate, 2008 [21] 2. Birrel et al., 2003 [22] 8. Dunlop et al., 2003 [29] 14. Johnson et al., 2008 [61] 20. Quintana et al., 2006 [59] 27. Zeni et al., 2010 [18].

11. Hawker et al., 2006 [26] 12. Hawker et al., 2000 [30] 13. Ibrahim et al., 2002 [40] 14. Johnson et al., 2008 [61] 15. Judge et al., 2010 [28] 3. Borkhoff et al., 2008 [55] 9. George et al., 2008 [20] 15. Judge et al., 2010 [28] 21. Riddle et al., 2009 [78].

16. Juni et al., 2010 [37] 17. Lievense et al., 2007 [19] 18. Linsell et al., 2005 [52] 19. Momohara et al., 2007 [23] 20. Quintana et al., 2006 [59] 4. Boutron et al., 2008 [60] 10. Hanchate, 2008 [21] 16. Juni et al., 2010 [37] 22. Schonberg et al., 2009 [46].

21. Riddle et al., 2009 [78] 22. Schonberg et al., 2009 [46] 23. Steel et al., 2008 [38] 24. Suarez-Almazor et al., 2005 [44] 25. Yong et al., 2004 [48] 5. Card et al., 2007 [47] 11. Hawker et al., 2006 [26] 17. Lievense et al., 2007 [19] 23. Steel et al., 2008 [38].

26. Zeni et al., 2008 [18] 6. Conner-Spady et al., 2008 [53] 12. Hawker et al., 2000 [30] 18. Linsell et al., 2005 [52] 24. Suarez- Almazor et al., 2008.

**Table 2:** Summary of quality of identified quantitative studies

**Study (lead investigator)**	**Subjects**	**Joint**	**Question/End point**	Score*	**Generalisable**
Borkhoff [55,56]	Primary care and surgeons	Knee	Referral	3	No(standardised cases)
Boutron [60]	Primary care doctors	Knee and hip	Referral	4	Yes
Quintana [57-59]	Specialists	Knee and hip	Recommendation	3	Yes
Birk [54]	Waiting list patients	Knee and hip	Decision to change surgeon	2	No
Birrell [22]	Primary care patients	Hip	Waiting list placement	2	Yes
Card [47]	Older adults	Hip and knee	Arthroplasty	2	Yes
Conner-Spady [27,53]	Waiting list patients	Knee and hip	Hypothetical change of surgeon	3	No
Cross [79]	Operated patients	Knee and hip	Willingness to pay	3	No
Dunlop [29]	Older adults	Knee and hip	Arthroplasty	2	Yes
George [20]	OA Medicare patients	Hip	Arthroplasty	3	No
Hanchate [21]	Older adults	Knee	Arthroplasty	3	Yes
Hawker [26]	OA patients	Knee and hip	Arthroplasty	4	Yes
Hawker [30-33]	OA patients	Knee and hip	Willingness to operate	3	Yes
Ibrahim [39-42]	Primary care males VA patients	Knee and hip	Willingness to operate	1	No
Johnson [61]	Primary care patients	Hip	Waiting list placement	1	Yes
Judge [28]	Older adults	Knee and hip	Arthroplasty	2	Yes
Juni [36,37]	Primary care patients	Knee and hip	Waiting list placement/Willingness to operate	1	Yes
Lievense [19]	Primary care patients	Hip	Arthroplasty	2	Yes
Linsell [52]	Retirement age adults	Hip and knee	Willingness to operate	2	Yes
Momohara [23]	RA patients	Knee	Arthroplasty	2	Yes
Riddle [78]	OA patients	Knee	Arthroplasty	2	No
Schonberg [46]	Retirement age OA patients	Knee and hip	Referral	2	No
Steel [38]	OA/RA patients	Knee and hip	Arthroplasty	3	Yes
Suarez-Almazor [44,45]	OA patients	Knee	Willingness to operate	3	Yes
Yong [48]	Non-obese retirement age adults	Knee	Arthroplasty	2	No
Zeni [18]	OA patients	Knee	Arthroplasty	2	Yes

*Sum of individual internal validity criterion rating (1 = study met criterion; 0 otherwise; maximum score: 4). Criteria: Prospective/longitudinal study and analysis; adjustment for confounding; reporting of attrition; doctor-assessed need.

### Studies on determinants of a patient’s decision to undergo surgery

#### Clinical status and quality of life

In end-stage knee OA (see Additional file 4, Additional file 5), patients with significantly weaker involved and uninvolved limbs, lower self-reported ability to function, or less knee extension were more likely to undergo TKA, whereas having bilateral as opposed to unilateral disease did not affect the probability of surgery [18]. Radiological information (Kellgren-Lawrence score greater than or equal to 2) and morning stiffness in a patient at an initial visit to a primary care doctor, each predicted his or her undergoing TKA within the subsequent three years. By six years after the initial visit radiological information, but not morning stiffness, predicted surgery (OR: 8.6; 3.0-24.6 [19]).

In patients with hip-related symptoms, difficulties with stooping and walking predicted primary THA within the following 3.5 years [20]. In addition to problems with getting up from a chair, climbing a flight of stairs, or crouching, stooping and walking difficulties each also predicted primary TKA within two years [21]. A diagnosis of cancer or diabetes reduced a patient’s likelihood of undergoing surgery, whereas diagnoses of heart disease, lung disease, or high blood pressure had no effect [21].

In a UK study, a scoring system comprising radiological parameters (internal rotation), hip pain severity, and the use of a walking stick enabled researchers to correctly identify 76% of primary care patients who were put on a waiting list for surgery, and 95% of those that were not, after 3 years [22]. Pain severity and functional disability were also independently, positively associated with primary TKA over a 5-year follow-up period in RA patients [23]. Pain is the single most important influential factor in the decision to undergo knee and hip arthroplasty [24], although patients define their need in terms of a doctor diagnosis based on x-ray test results [25]. Patients with test scores indicating more severe arthritis (WOMAC), or higher health status (SF-36 General Health score), were predisposed to undergo primary TKA or THA [26], while patients with higher quality of life scores (EuroQol 5-Dimension, EQ-5D; see Additional file 5) expressed less willingness to change surgeons in order to reduce operation waiting time [27].

#### Age

**Figure 2:**
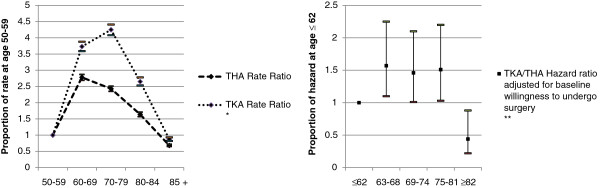
**Ratios: a) age ≥ 60 to age 50–59 surgery rates (left) and b) propensity for surgery at age > 62 relative to age ≤62 rate (right).** * Rates in public hospitals in England, adjusting for gender, ward ethnic mix and deprivation, distance to and characteristics of hospitals [28]. ** Rate of receipt of primary surgery after five years, based on population samples from two areas of Ontario, Canada. Relative to age ≤ 62. Adjusting for WOMAC, SF-36 General Health, Willingness to undergo surgery [26].

Age is a complex factor in predicting whether patients will undergo TJA, since their willingness to do so can depend on their expectations of functionality, which change with age. Among a group of patients visiting their primary care doctor with chronic hip pain, those aged 60 years or older were more likely to undergo THA within three and six years [19]. In England, the rate of surgery has been observed to increase with age to a peak at 60–69 for THA, and at 70–79 for TKA, before declining to a nadir at age ≥80 [28]. Likewise for the United States, octogenarians have reportedly lower rates for primary THA [20], and for TKA or THA overall [29]. That patients aged 62 years or younger and those in their eighties are less likely to receive TJA is confirmed by the only study that controlled for willingness to undergo surgery (and, implicitly by its taking place in a public health system, for economic access), which also found no apparent variation in the incidence of TKA or THA among those in the age range from 63 to 81 [26] (Figure 2b). In fact, a precursor to the study had found those older than 65 to be less willing to operate [30-33]. Studies from the United States have discrepant results, suggesting greater use after age 60, which in two cases may be difficult to interpret since the studies controlled neither for health insurance status nor income [18,19]. On the other hand, the third was the only instance of a study controlling for employment status and economic access (i.e., health insurance), and found that individuals under 65 were less likely to undergo TKA than older adults (OR: 0.72; 0.52-1.01) [21]. Thus, while functional expectations and perceived need vary inversely with age [25,34], it appears that some of the lower utilisation rates of the youngest and oldest patients in need (Figure 2a) are due to limited access to surgery [34]. 

#### Gender

Women view unmet functional needs as primarily associated with walking and shopping limitations, whereas men discuss their effects on leisure activities [25]. Women older than 54 experienced higher levels of unmet need for arthroplasty, but had lower rates of surgery than men of the same age. While more likely to seek treatment for their arthritis given similar self-reported comorbidities, women were less likely to have discussed the option of arthroplasty with a primary care doctor [30-33]. Contrary to reports that women are more risk averse and undergo surgery later [35-37], no gender difference was found in terms of willingness to undergo treatment, which predicted surgery within five years [26]. These results led researchers to propose the doctor-patient interaction as the source of barriers to surgery [30-33].

**Figure 3:**
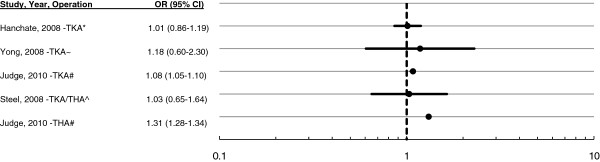
**Ratio of male to female surgery rate.** *U.S. population-based study age ≥ 47. Patients with an arthritis-related visit to the doctor in the past two years at baseline. Adjusted for age, gender, race, comorbidities, functional limitations, income, wealth, insurance type, employment, and BMI ≥ 25 [21]. ~ Rate over 18 months, from south of England study of patients aged ≥65. Unadjusted for confounders [48]. #Surgical rates in public hospitals, population of England. One year incidence, adjusted for socio-economic and ethnicity mix of ward of residence [28]. ^Respondents reporting an arthritis-related visit at baseline. Adjusting for demographics, health need and economic access (including health insurance, wealth and education) [38].

Gender had no effect on patient receipt of primary TKA or THA in Toronto, Canada [26], or in the United States [18,20]. Similarly, gender did not affect the two-year rates of respondents to the U.S. Health and Retirement Study (HRS) [21,38] or among members of an elderly cohort [29]. Only one study reports a gender differential: underutilisation by women that is larger in the more deprived districts of England (Figure 3) [28]. However, the study’s limited control for confounders, relying as it does on deprivation and ethnicity mix measures at the ward level to proxy individual respondent socio-economic status, casts doubt on the validity of its findings (Additional file 4). As for waiting-list patients, men have been more likely than women to respond affirmatively to a hypothetical question about changing to an equally qualified surgeon with a shorter waiting time [27]. 

#### Race

**Figure 4:**
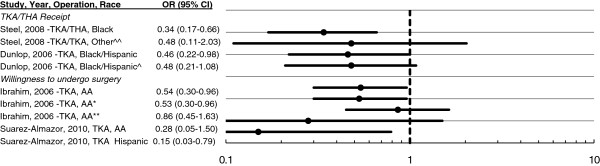
**Ratio of surgical rate and willingness to undergo surgery by race minority group to white.** Dunlop: Adjusting for demographics, health need and economic access (including health insurance, wealth and education). Black/Hispanic refers to all respondents; Black/Hispanic^ refers to respondents reporting an arthritis-related visit at baseline [29]. Steel: Adjusting for covariates age, gender, wealth, employment, BMI ≥ 30, seen doctor ≥2 times in last 2 years, education, comorbidity, grandchild care, difficulty walking 1 block or more, married/cohabiting. Other category is defined as non-black and non-white [38]. Ibrahim: AA African-American sample adjusting for age, level of education, annual income, radiologic severity of disease, WOMAC, geriatric depression score; AA* Adjusting for familiarity with surgery in addition to covariates for AA[40]; AA** Adjusting for familiarity and expectations in addition to covariates for AA [40]. Suarez-Almazor: Adjusted for age, gender, years of education, trust in physician, perception of efficacy, perception of risk, WOMAC, relative/friend with TKA. AA refers to African American sample [44].

Evidence on this factor was found only for the United States (Figure 4). There are documented disparities in two-year rates of hip and knee surgery for arthritis between blacks (2% per year), Hispanics (1.79%), and whites (4.35%) in a national cohort of persons older than 69 [29] that are robust to adjustment for demographic, health, and economic access factors, including health insurance. Together with results on hip and knee surgery for HRS respondents older than 59 [38], these findings suggest that ethnic minorities have lower utilisation of TJA independent of access factors. 

In addition to differences in disease severity, comorbidity, and socio-economic characteristics, minorities also have a lower willingness to undergo the operation. This inclination is due to lower expectations of surgical efficacy and less familiarity with the operation among African Americans, who are less likely to know someone treated with arthroplasty, more likely to think it involves extended hospitalisation and recovery and more likely to have concerns about possible outcomes (Figure 4, bottom half) [39-45]. Nevertheless, severe OA patients aged 65 and older who discussed surgery with their primary care doctors were more likely to undergo surgery in the following year, and the likelihood of having such a discussion did not differ between white and non-white patients once the effects of education and income were accounted for [46]. 

**Table 3:** Specialist referral and waiting list prioritisation instruments, levels and scores for THA and TKA

CAHTA*			CPAC**			WCWLP***		
**Criteria**	**Level**	**Score**	**Criteria**	**Level**	**Score**	**Criteria**	**Level**	**Score**
**Severity of disease (clinical and radiological exploration)**	Moderate	0	**Pain on examination**	None	0			
	Severe	18		Mild	6			
				Moderate	15			
				Severe	30			
			**Other abnormal findings**	None	0	**Abnormal findings on physical exam**	None/mild	0
				Mild	2		Moderate	5
				Moderate	5		Severe	9
				Severe	10			
			**Multiple joint involvement**	No	0			
				Yes, affected joints with moderate severity	4			
				Yes, severe involvement	10			
**Pain**	Mild	0	**Degree of pain**	None	0	**Pain on motion**	None/mild	0
	Moderate	17		Mild	4		Moderate	13
	Severe	33		Mild to moderate	6		Severe	20
				Moderate	9			
				Moderate to severe	14			
				Severe	20	**Pain at rest**		
**Probability of recovery**	Moderate	0						
	High	4						
**Difficult in doing ADL**	Some difficulty	0	**Time walked**	Unlimited	0	**Ability to walk without significant pain**	Over 5 blocks	0
	Great difficulty	10		31-60 min.	2		1-5 blocks	4
	Unable to do most ADL	20		11-30 min.	4		Less than 1 block	8
				2-10 min.	6		Household ambulatory	13
				2 < min. or indoors only	8			
				Unable to walk	10			
			**Other functional limitations**	None	0	**Other functional limitations (ADL)**	None	0
				Mild	2		Mild	2
				Moderate	4		Moderate	4
				Severe	10		Severe (unable to do most activities)	10
**Limitation on ability to work**	No/does not work	0	**Ability to work, give care to dependents or live independently**	Not threatened or difficult	0	**Ability to work, give care to dependents or live independently**	Not threatened but more difficult	0
	Yes	10						
				Not threatened but more difficult	4		Threatened but not immediately	7
**Has someone to look after the patient**	Yes	0						
	No	9		Threatened, but not immediately	6		Immediately threatened	10
**Be a care-giver**	No	0						
	Yes	6		Immediately threatened	10			

*Catalan Agency for Health Technology Assessment [62]; **Clinical Priority Assessment Criteria [63].***Western Canada Waiting List Program [65] (asks about situation over the past three months).

#### Education

**Figure 5:**
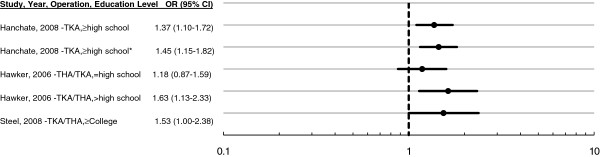
**Ratio of surgical rate with post-secondary education to rate with lower education level.** Hanchate: Relative to less than high school education. U.S. population-based study age ≥ 47. Adjusted for age, gender, race, comorbidities, functional limitations, income, wealth, insurance type, employment, BMI ≥ 25. *Refers to respondents with an arthritis-related visit to the doctor in the past two years at baseline; other result refers to all respondents [21]. Hawker: Relative to less than high school education. Sample from two areas in Ontario, Canada. Adjusting for WOMAC, SF-36 General Health, age [26]. Steel: Relative to less than college education. U.S. population-based study age ≥ 60. Adjusting for covariates age, gender, wealth, employment, BMI ≥ 30, seen doctor ≥2 times in past 2 years, comorbidity [38].

Three studies report the effect of education on the probability of surgery. Post-secondary education is associated with higher likelihood of primary surgery, whether to treat knee OA [21], knee or hip OA [38], or for patients in need of primary TKA or THA surgery (Figure 5) [26]. On the other hand, a study among 198 OA patients in the United States found that education had no effect on the probability of considering TKA in the event of the knee arthritis worsening and the doctor recommending surgery, by controlling for perception of efficacy and race [44]. Other U.S. studies of TKA or THA and of THA [20,29], report ‘statistically insignificant’ results without providing the estimates. Post-secondary education has also been positively associated with patients’ willingness to change surgeons in order to reduce their waiting time for surgery (OR: 1.73, 1.15-2.62 [27]). 

#### income/health insurance

In the United States, lack of health insurance under the age of 65, low household income per cohabitant, and household assets below $5,000 (in 1998 prices) were independently associated with low TKA use [21]; by the age of full retirement, health insurance and multiple insurance plans increased the likelihood of TKA or THA [47]. Supplemental health insurance also increased operation rates in the Medicare population (OR: 0.46; 0.22-0.95)[29]. In Toronto, Canada, where universal public health insurance exists, income had no effect on primary TKA or THA, after adjusting for education [26]. By contrast, low income was associated with a low probability of patients in northeast England having gone through surgery within 18 months of their initial visit to a primary care doctor with a complaint of knee pain [48], but this evidence is of limited value since the study failed to control for confounders.

#### Employment

Using HRS data for the period 1996–2004, Hanchate and colleagues [21] estimated a higher two-year incidence of TKA with current employment relative to no employment (OR: 1.28; 1.04-1.58), after adjusting for relevant confounders, including age under 65. A separate independent analysis of the probability of TKA or THA, among HRS respondents older than 59 during the period 1998–2004 resulted in a similar but imprecise estimate (OR: 1.29; 0.74-2.25)[31], which also differed from the first analysis in its lack of adjustment for health insurance status. Paid employment had no independent effect on time to first TKA or THA in an Ontario, Canada, cohort of persons with OA aged 55 and older [26].

#### Other Factors

**Figure 6:**
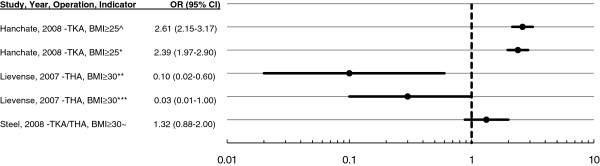
**Ratio of surgical rate overweight (BMI ≥ 25) or obese (BMI ≥ 30) to rate with a lower BMI.** ^U.S. population-based study age ≥ 47. Patients with an arthritis-related visit to the doctor in the past two years at baseline. Adjusted for age, gender, race, comorbidities, functional limitations, income, wealth, insurance type, employment, and education [21]. *Arthritis subsample. Other details as in Footnote ^. **Three-year rate. Adjusted for age, morning stiffness, Kellgren-Lawrence ≥ 2, worst pain location, function with decreased active hip motion [19]. ***Six-year rate. Other details as in Footnote **. ~U.S. population-based study age ≥ 60. Adjusting for covariates age, sex, wealth, employment, seen doctor ≥2 times in past 2 years, education, and comorbidity [38].

Caring responsibilities may discourage eligible older patients from taking up arthroplasty [30]. Self-awareness of weight problems has also been found to discourage patients from undergoing surgery [34]. Furthermore, being overweight or obese appears to reduce the likelihood of subsequent use of THA [19], although it does not have the same effect on use of TKA [21,38] (Figure 6). 

When assessing suitability for surgery, specialists take into consideration the patients’ preferences on leisure activities, as well as work, and family life circumstances, including cohabitation [49]. Those waiting for TKA often worry about becoming reliant on family and friends for their daily activities [25], and women have recorded higher levels of unmet need and disability than men, primarily because proportionally more of them live alone [30]. By contrast, one study found that the civil status of Medicare OA patients had no effect on their probability of undergoing primary THA [20]; however, the result may have lacked statistical power.

Hip and knee OA patients who end up being referred to specialists either seek care early on with the view toward preventing their disease from becoming worse or wait until symptoms become unbearable to seek treatment [45,50]. Their decisions to undergo surgery are prompted by increasing severity of limitations that affect their basic quality of daily living, relationships, and psychological well-being [51]. Contrary to other independent results [52], an analysis controlling for race and perceived severity found that having a friend or relative who had undergone TKA had no effect on the willingness of persons with knee OA to consider surgery. However, this result and the equally absent evidence of effect for perceived risk level [44] may be due to the small size of the respective study (n = 198).

Indeed, qualitative evidence suggests that patients are susceptible to influence from the negative surgical experiences of other persons [30,45], and that their preferences on TKA are also influenced by previous personal experience of surgery, including non-orthopaedic types of surgery [45]. In southern England, individuals with chronic knee pain were more likely to have consequently sought treatment from their GP than were persons with chronic hip pain, and were less [36] or equally likely [52] to have been referred to and seen a specialist. In these studies, knowing others who had had surgery was associated with positive attitudes about surgical success and TKA patients had fewer such acquaintances than THA ones did.

Differences in healthcare access between hip and knee patients have occurred at the referral stage in both primary [36] and secondary care [52]. Moreover, across English districts, a study has reported replacement rate variations from 1.22 to 14.4% for hips and from 1.05 to 6.19% for knees. While rural areas tended to have greater use of hip replacement, knee replacement was more common in urban settlements [28].

Studies of hypothetical choices, in Canada [27,53], and actual decisions, in eastern Denmark [54], found that, when patients were presented with the opportunity to change surgeons of comparable quality in order to undergo surgery earlier, 50‐‐60% of patients declined it. Having an expressed preference for a specific doctor reduced the odds of changing surgeons by 43%, while having a certain date of operation dominated waiting time considerations.

Patients who declined re-referral from a local hospital to one beyond their county of residence also declared the longer distance and transport time as main reasons for their decision [54].

### Studies with surgeons and general practitioners

In a study in Ontario, Canada, prior discussion of TKA or THA with a physician emerged as the driver of patient willingness to consider surgery [30]. Hypotheses about gender disparities in the contents of such patient-doctor interactions [30] are supported by results from another Canadian study of GPs and orthopaedic surgeons’ blinded referrals or recommendations of TKA for two standardised moderate knee OA patients who differed only in gender [55,56]. GPs referred men to specialists 1.3 times as often as they referred women, while surgeons were nine times as likely to recommend surgery to men as they were to women. On the other hand, a cross-sectional study in the United States found no differences in the likelihood of a physician recommendation of surgery across ethnic groups after adjusting for age, gender, education and disease severity [44]. Consistent with these results, in a study in 40 general practices in southwest England, their referrals to specialists, consultations with orthopaedists, and waiting list assignments for hip replacements were less frequent for women than for men. These findings were robust to adjustments for willingness to undergo surgery and other covariates, despite the fact that no differences between genders had been found in patients’ access to drug therapy for chronic hip pain [37]. In this regard, it is worth noting that doctors rely on non verbal signs, such as perceived tiredness suggestive of severe night pain, or anxiety in a patient attending consultation, to decide on the manner and timing of communicating information about surgical risk [49].

As for health specialist opinions, the likelihood of classifying an individual THA case as appropriately referred was most influenced by pain and, secondly, by functioning; bone quality was of marginal significance [57]. Six months after THA, patients classified as ‘appropriate’ and ‘uncertain’ had lower complication and mortality rates, and similarly higher improvements in the physical and pain domains of the SF-36 and the three dimensions of the WOMAC than patients classified as ‘inappropriate’. For classifying TKA patients, symptoms took precedence, followed by radiological evidence, mobility, age, previous surgical management, and localisation [58]. ‘Appropriate’ TKA referral patients saw greater gains in the social function score and the three WOMAC dimensions than ‘inappropriate’ patients [57,59].

In France, a GP’s opinion that an OA patient would need surgery within the following 12 months was primarily determined by clinical factors, with severity of disease the most influential determinant. Cases analysed in this study had more days with pain, were more disabled (Lequesne and WOMAC; Additional file 3), and had lower levels of health-related quality of life (SF-36 PCS and MCS; Additional file 5) than other patients. They were more likely to live in a rural environment, often male, older, and took medication for OA more often. The only determinant that varied between hip and knee conditions was gender, which affected only hip patients [60].

In England, an Oxford Hip Score greater than or equal to 34 and radiographic evidence of a complete loss of joint space or severe marginal osteophyte formation together correctly predicted 87.5% of cases deemed by surgeons to require THA and 50% of cases deemed not to require THA [61]. Prioritisation of patients for surgery based on doctors’ opinions moderately reflected the views of patients about the difficulty caused by their condition, while strongly reflecting the views of orthopaedic surgeons about their patients’ priority for surgery (see Table 3[62]). Priority is given to those with severe clinical and radiological disease, more severe pain, high probability of recovery, greater difficulty in performing activities of daily living, and to patients affected in their ability to work, without a caregiver, and with caring responsibilities for another person. Among general practitioners, similar assessments are used for rationing in New Zealand [63,64] and Canada [65].

A study of English general practitioners by Linsell and colleagues documents lower utilisation of x-ray tests and similar referral rates for primary care patients with chronic knee pain relative to patients reporting hip pain [52]. Consequently, the authors argue that GPs in Oxfordshire, appear to follow national guidelines, which state that knee OA is best diagnosed clinically, contrary to the recommended practice for hip OA [66]. However, there are examples where the availability of radiological information determined the likelihood of referral to specialists in both hip and knee patients [19,22]. Moreover, in-depth interviews of specialists have revealed that the decision to offer surgery involves “various judgements and skills derived from experience” which are partly “instinctive and partly informed by the literature” [49].

## Discussion

This review summarises the evidence on determinants of hip and knee replacement in patients eligible for the operation. Across all the studies assessed for this review, the likelihood of a patient receiving arthroplasty depended mostly on clinical characteristics of the joint, physician recommendations, patients’ perceptions and preferences, and interactions between doctors and their patients. In public health systems, willingness to undergo surgery was the most important determinant of receipt, implying that the effect on surgery receipt of patient characteristics represents primarily behavioural variation by patients rather than by doctors acting on their behalf. Underpinning demand for THA and TKA were education and, in the United States, possession and extent of health insurance coverage.

Some studies analyse predictors of surgery without accounting for socio-economic determinants, which are particularly important in health systems without universal public healthcare [32]. Socio-economic differences account for most of the gender- and age-related variations in utilisation, but not for those related with race. In the United States -the only country for which the question of racial disparities was addressed- racial minorities were less willing to undergo surgery due to their low expectations for surgical outcomes. Such differences in expectations appear to be determined by social networks [67] and culture surrounding minority groups, and point to questions about the quality of healthcare available to them [68]. These observations, together with the reviewed evidence on larger gender-related gaps in more deprived regions of England, suggest that underutilisation of healthcare technology may be socially, economically, and culturally determined.

The age of prospective patients may have an impact on their willingness to undergo surgery. In the range from 50 to 70 years of age, patients’ willingness appears to decline with increasing age. However the extent to which this effect depends on retirement opportunities of patients or their partners or availability of informal care by friends and relatives is unclear, as no study has addressed the issue. One study reports an association between TKA receipt and employment suggesting that TKA is valued more by persons engaged in paid work than by other individuals with the same socio-economic status, health insurance type, education, disease, health status, and demographic characteristics [21]. A second study, found the same magnitude of effect on TKA or THA but, unlike the first, was confounded by the lack of adjustment for health insurance status [38]. Since health insurance in the United States is positively correlated with arthroplasty [47] and negatively correlated with employment two years subsequently [69], the study is likely to underestimate the combined effect on THA or TKA, thus suggesting that employment has a stronger effect on THA than that reported for TKA [21]. Further research is warranted to establish whether a causal relationship running from employment to TJA use exists. If so, additional analysis may be required to understand whether the nature of such relationship originates from economic incentives for patients to demand healthcare [70], or inducement by doctors [71,72]–as would be the case, for example, if surgeons in the United States were to be more inclined to recommend surgery to VA members with supplementary insurance than to those with only VA coverage [42]. A third paradigm would consider also the influence on decision making of social norms and identities [73].

Studies on clinicians’ views have found that primary care doctors and orthopaedic surgeons hold different opinions about patient eligibility for surgery [10]. Primary care doctors generally want patients to meet higher thresholds of disease severity than those required by orthopaedic surgeons. It has been suggested that primary care doctors may lack adequate information about the risks and benefits of total joint replacement, and may therefore inadvertently restrict access to healthcare [2]. Nevertheless, the decisions of primary care doctors about relative healthcare service use and referrals in the south of England were found to be consistent with OA management guidelines, which recommend that patients with knee pain be diagnosed on the basis of clinical rather than radiological evidence [52]. These guidelines for managing patients with knee symptoms are contrary to the guidelines for managing patients with hip symptoms, but there is evidence that radiographic evidence determines referrals and receipt of surgical treatment for both knee and hip patients [74].

The interactions between primary care physicians and their patients matter for increasing access to surgery. Prior discussion of arthroplasty with a physician may favourably influence patients’ willingness to undergo surgery. However, women appear to have a lower chance of being referred to secondary care and receiving a specialist’s recommendation for TJR. The latter finding merits further study, especially in view of evidence that specialists rely on informal judgment and inference when deciding whether to offer surgery. Moreover, the magnitude of geographical variations in utilisation in England [28] and the United States [75], which persist over many years [75], may not be plausibly explained by corresponding variations in patient preference and need, but appear to be driven by variations in local medical practice associated with differences in established professional opinion [76]. A study of racial and ethnic disparities of knee replacement rates in the United States has estimated that 38% of the gap of black relative to white women was accounted for by hospital referral region of residence [77].

Despite the inherent uncertainty of surgical decisions and the lack of specific guidelines, patients are regularly given priority for surgery based on their limitations in paid work and caring responsibilities. However, it is not known what relative independent importance these factors have for priority of access, nor their relevance across health systems with varying rates of population health insurance coverage. Research on these issues would help to elucidate the relationship between women’s differential use of TJA and gender inequality in the labour market.

The current gaps in evidence suggest the need for a combination of research designs, including observational prospective longitudinal studies in patient cohorts, secondary data analysis of general population surveys of older patients, and experimental preference studies, to investigate the relevant facets of decision making in TJR. Some of these studies are already in progress [78], or may soon follow on the experience of precursors [21,79]. Indeed, much of the existing literature that did not qualify for the present review [80-104] may inform their design. It is worth noting that heterogeneous research methods have hampered the consistent accumulation of evidence, particularly in relation to measuring patient need or severity of disease [105-110] and consequently defining the population of those patients who are able to benefit from surgery.

## Conclusion

Access to hip and knee arthroplasty is driven by patients’ willingness to undergo surgery. In turn, patients’ willingness is determined by their knowledge about the technology and their expectations about the outcomes of the procedure, and by local or regional idiosyncratic surgical decision making practice. There is significant variation in patient willingness around the age of retirement from the labour market, but no study has investigated the effects of retirement plans or economic incentives to retire for patients or their partners, on whether or when to undergo an operation. A study is also needed to learn about the difference in the importance of such considerations between men and women. Research on these questions would inform planning to provide for the healthcare needs of an ageing population with a longer working lifespan.

## Competing interests

The authors declare no competing commercial or non-commercial interests.

## Authors’ contributions

RM designed the study and its data collection forms, screened and reviewed studies and extracted the data, and wrote the manuscript. RT contributed to writing the manuscript and its design. OC screened and extracted the data. JB contributed to writing the manuscript. MD contributed to designing the study and writing the manuscript. All authors read and approved the final manuscript.

## Pre-publication history

The pre-publication history for this paper can be accessed here:

http://www.biomedcentral.com/1472-6963/12/225/prepub

## Supplementary Material

Additional file 1 **Appendix: Search Strategy.** This appendix presents the electronic search strategy employed to retrieve study records in Medline (Ovid) and Embase. Click here for file

Additional file 2 **Table S1.** Characteristics of Included Studies. This table describes the individual studies reviewed, including study year, country, setting, population of subjects, design and follow-up, and measure of need used. Click here for file

Additional file 3 PRISMA 2009 Checklist. Click here for file

Additional file 4 **Table S2.** Results of Quantitative Studies. This table contains the detailed results presented by individual studies alongside the summary characteristics of the study sample. Readers may refer to this file if they want to learn about the statistical details of the individual study findings. Click here for file

Additional file 5 **Table S3.** Disease Specific and Generic Health-Related Quality of Life Outcome Tools). This file presents a summary table describing the health related quality of life tools used by the studies reviewed. This information may help readers to interpret the detailed results in Additional file 4. Click here for file
