# Supplementary material for: Determinants of demand for total hip and knee arthroplasty: a systematic literature review
Source: BMC Health Serv Res. 2012 Jul 30;12:225. doi: 10.1186/1472-6963-12-225 (PMC3483199; doi:10.1186/1472-6963-12-225)
Supplement: Additional file 1 — Appendix: Search Strategy. This appendix presents the electronic search strategy employed to retrieve study records in Medline (Ovid) and Embase. [file 1472-6963-12-225-S1.docx]

**Search Strategy**

Literature Search (last updated 6 September 2011)

The Ovid (Medline) search strategy was the following:

#1 Arthroplast$.mp. or Arthroplasty, Replacement/ or Arthroplasty, Replacement, Knee/ or Arthroplasty/ or Arthroplasty, Replacement, Hip/ or ((hip or knee) adj3 prosthes#s).mp. (51740)

#2 "Patient Acceptance of Health Care"/ or Willingness.mp. or * "Physician’s practice patterns" (52950)

#3 "Health Services Needs and Demand"/ or demand.mp. (80121)

#4 2 or 3 (131040)

#5 undergo.mp (89159)

#6 5 or 4 (219051)

#7 1 and 6 (1084)

#8 limit 7 to (English language and humans) (957)

Of these 57 hits were duplicates.

The Embase search strategy involved the terms:

#1 arthroplast*:ab,ti OR replacement:ab,ti AND [embase]/lim 163,026

#2 hip:ab,ti OR knee:ab,ti AND [embase]/lim 126,146

#3 'patient acceptance of health care'/exp/mj OR ‘patient acceptance of health care’ OR willingness:ab,ti OR undergo:ab,ti OR ‘clinical practice’/exp/mj AND [embase]/lim 133,766

#4 #1 AND #2 31,240

#5 'health services needs and demand'/exp/mj OR ‘health service needs and demand’ OR demand:ab,ti AND [embase]/lim 492,854

#6 #3 OR #5 605,656

#7 #4 AND #6 1,444

#8 #7 AND [humans]/lim AND [english]/lim 1,067

Of these, 310 were duplicate records or records already found in the Ovid search
