# Supplementary material for: Determinants of demand for total hip and knee arthroplasty: a systematic literature review
Source: BMC Health Serv Res. 2012 Jul 30;12:225. doi: 10.1186/1472-6963-12-225 (PMC3483199; doi:10.1186/1472-6963-12-225)
Supplement: Additional file 2 — Table S1. Characteristics of Included Studies. This table describes the individual studies reviewed, including study year, country, setting, population of subjects, design and follow-up, and measure of need used. [file 1472-6963-12-225-S2.docx]

**Table ii Results of quantitative studies**

| **STUDY ID** | **SAMPLE CHARACTERISTICS** (Percentages unless otherwise stated) | **MULTIVARIATE ANALYSIS RESULTS** |
| --- | --- | --- |
| Birk, et al., 2006[54] | \| **Variable** \| Replied to invitation  (N=125) \| Not replied to invitation (N=19) \| \| --- \| --- \| --- \| \| Age, mean (SD) yrs \| 64.6 (11) \| 66.6 (14) \| \| Male sex \| 56 \| 84 \| \| Hospital A \| 38 \| 42 \| \| Hospital B \| 62 \| 58 \| \| Hip \| 69 \| 68 \| \| Knee \| 31 \| 32 \| \| Accept \| 61 \| 53 \| \| Decline \| 39 \| 47 \| | Acceptance of re-referral to shorter waiting lists   \| **Variable** \| OR (95 % CI) \| \| --- \| --- \| \| Hospital (C/B) \| 1.91 (0.85-4.32) \| \| Acceptable waiting time to surgery \| 1.39 (0.57-3.39) \| \| Age \| 0.82 (0.41-1.65) \| |
| Birrel, et al., 2003[22] | \| **Variable** \| Patients with hip pain  (N=195) \| \| --- \| --- \| \| Female sex \| 68 \| \| Age mean, years (SD) \| 63 (11) \| \| Side of pain: Right \| 53 \| \| Left \| 45 \| \| Both \| 3 \| \| Range of movement median (IQR):Flexion \| 98° (84-110) \| \| Internal Rotation \| 26° (20-34) \| \| External Rotation \| 28° (20-35) \| \| Pain VAS score, median (IQR) \| 50 (30-70) \| \| Pain duration: Less than 3 months \| 30 \| \| 3-12 months \| 41 \| \| More than 12 months \| 28 \| \| Reported previous episodes of hip pain \| 38 \| | Study stated that when clinical variables were included, restriction in internal rotation, pain score, pain duration, and use of a walking stick were all independent predictors from forward stepwise selection Cox proportional hazard regression analysis.  When both clinical and radiographic predictors were included in the regression analysis, radiological variables, namely, minimum joint space, and Croft grade, and the use of a walking stick and the pain score were independent predictors of being put on a waiting list. No other details on results were given. |
| Borkhoff et al., 2008 [55,56] | \| **Variable** \| All physicians  (N=67) \| Orthopaedic surgeons (N=29) \| Family physicians (N=340) \| \| --- \| --- \| --- \| --- \| \| Male \| 82.0 \| 90.0 \| 76.0 \| \| Age yr, mean (min-max) \| 52(33-77) \| 52(38-67) \| 52(33-77) \| \| Years in practice, mean (min-max) \| 21(2-49) \| 21(5-35) \| 21(2-49) \| \| Practice type: Solo  Group  University affiliated  Community practice \| 70  30  34  66 \| 90  10  41  59 \| 55  45  29  71 \| \| Estimated no of OA patients seen in 1 month, mean (min-max) \| 36(3-200) \| 53(10-200) \| 21(3-100) \| | ***Physicians’ recommendations for TKA and sex of standardized patient, by physician type***   \| Physician type \| OR (95% CI) \| RR (95% CI) \| \| --- \| --- \| --- \| \| Orthopaedic surgeons (n=29) \|  \|  \| \| Female patient \| 1 \| 1 \| \| Male patient \| 22.1(6.4-76.0) \| 9.0 (3.0-26.9) \| \| Family physicians (n=38) \|  \|  \| \| Female patient \| 1 \| 1 \| \| Male patient \| 2.21 (1.04-4.71) \| 1.35 (1.01-1.81) \|   Note: this analysis involved standardised patients, and thus implicitly controls for differences (other than sex) |
| Boutron et al., 2008 [60] | \| **Variable** \| No indication  (N=3272) \| Indication (hip/knee OA) (N=849) \| \| --- \| --- \| --- \| \| Age mean ( SD) \| 67 (9) \| 70 (9) \| \| Age ≥65 \| 5 \| 72 \| \| Male sex \| 41 \| 43 \| \| Living: alone  In institution  With partner/family \| 60  1  39 \| 30  2  68 \| \| High school/ Postgraduate education \| 44 \| 36 \| \| No work during last year \| 77% \| 8 % \| \| Lequesne score 0-24, mean (SD) \| 11.2 ( .1) \| 14.9 (3.6) \| \| SF-36 PCS (0-100)  SF-36 MCS (0-100) \|  \|  \| \| Years OA duration, mean (SD) \| 5.5 (5.0) \| 6.0 (4.5) \| | ***Factors in GPs’ indication for TJR within 1 yr post consultation***   \| **Variable** \| OR (95 % CI) \| \| \| --- \| --- \| --- \| \| Hip \| Knee \| \| Lequesne score (> severity threshold) \| 2.62 (1.83-3.75) \| 2.96 (1.71-4.25) \| \| ≥10 days with pain per month \| 1.95 (1.50-2.55) \| 1.39 (1.10-1.75) \| \| ≥10 days with disability per month \| 1.39 (1.07-1.82) \| 1.50 (1.19-1.91) \| \| Patients’ opinion of their disability (moderate/severe) \| 1.74 (1.20-2.51) \| 1.57 (1.01-2.20) \| \| SF-36 PCS \| 0.96 (0.94-0.99) \| 0.97 (0.95-0.99) \| \| Rural Environment \| 1.49 (1.09-2.04) \| 1.43 (1.09-1.87) \| \| Age≥65 \| 1.51 (1.07-2.11) \| 1.87 (1.36-2.56) \| \| Gender (male) \| 1.92 (1.39-2.65) \| 1.18 (0.71-1.47) \| \| Treatment for OA (Yes) \| 2.89 (0.92-9.04) \| 2.22 (0.85-5.82) \| |
| Conner-Spady et al., 2008[27] | \| **Variable** \| Patients waiting for TJR (N=557) \| Patients post TJR (N=643) \| \| --- \| --- \| --- \| \| Age mean ( SD) \| 69.2 (11) \| 70.5 (11) \| \| Male sex \| 45 \| 61 \| \| Married or with partner \| 68 \| 69 \| \| Living alone \| 24 \| 26 \| \| Education (completed high school or higher) \| 79 \| 78 \| \| Urban residence \| 52 \| 49 \| \| First surgery \| 70 \| 66 \| \| EQ-5D, mean (SD) \| 11.2 (4.1) \| 14.9 (3.6) \| \| EQ-5D VAS, mean (SD) \| 56.5 (20) \| 71.9 (17) \| \| Hip Replacement \| 52 \| 50 \| \| Waiting time mo, median (IQR) \| 8.0 (4.6-13.4) \| 8.3 (3.7-15.5) \| \| Had a preference for surgeon \| 36 \| 37 \| \| Given option of a surgeon \| 42 \| 42 \| | ***Likelihood of changing surgeons to one with a shorter waiting time for TJR***   \| **Variable** \| OR (95 % CI) \| \| --- \| --- \| \| Acceptable Waiting time to see surgeon \| 0.50 (0.36-0.70) \| \| Acceptable waiting time to surgery \| 0.62 (0.43-0.91) \| \| Age \| 0.98 (0.97 -1.00) \| \| EuroQol (EQ-5D) Index \| 0.39 (0.24 -0.66) \| \| Education \| 1.73 (1.15 -2.62) \| \| Perceived fairness regarding waiting time \| 0.53 (0.36 -0.78) \| \| Group \| 1.71 (1.19 -2.46) \| \| Male sex \| 1.49 (1.10 -2.02) \| \| Surgeon preference \| 0.57 (0.42 -0.79) \| \| Waiting time \| 1.00 (0.98 -1.02) \| |
| Cross et al., 2000 [79] | \| **Variable** \| THA (N=109) \| TKA (N=129) \| \| --- \| --- \| --- \| \| Response rate \| 77 \| 72 \| \| Age mean ( yr) \| 67.2 \| 73.5 \| \| Male sex \| 53.2 \| 40.3 \| \| Mean time since replacement (yr) \| 2.89 \| 2.84 \| \| Employed (FT/PT/casual) \| 33.3 \| 8.1 \| \| Receive pension \| 38.0 \| 68.5 \| \| Have private health insurance \| 76.1 \| 58.7 \| \| Report having other medical condition \| 51.0 \| 71.0 \| | ***Predictors of (any) WTP (ex-post)***   \| **Variable** \| OR TKA (N=102) \| OR THA (N=102) \| \| --- \| --- \| --- \| \| Age \| 1.14 (1.03-1.25) \| NS \| \| Male sex \| NS \| NS \| \| Income UAS$20,000 or more \| NS \| 19.87 (2.2 – 179.3) \| \| Private health insurance \| 12.61 (2.43-65.31) \| NS \| \| Recommend Replacement \| 42.88 (3.05-602.5) \| NS \| \| WOMAC Pain \| 0.80 (0.68- 0.94) \| 0.75 (0.62- 0.92) \| \| SF-36 General Health \| NS \| NS \| \| SF-36 Social Function \| NS \| NS \|   NS: Only stated as not significant (i.e. p>0.05) |
| Dunlop et al., 2003 [29] | \| **Variable** \| White  (N=5007) \| Black (N=812) \| Hispanic (N=340) \| \| --- \| --- \| --- \| --- \| \| Male \| 37.6 \| 32.5 \| 38.0 \| \| Age 70-79 (youngest group) \| 69.8 \| 66.6 \| 69.3 \| \| Arthritis \| 25.8 \| 42.8 \| 46.0 \| \| Other chronic conditions \| 59.8 \| 76.5 \| 69.4 \| \| Life threatening conditions \| 48.7 \| 37.0 \| 34.5 \| \| Any physical limitation \| 54.1 \| 65.0 \| 63.9 \| \| IADL limitations only \| 9.6 \| 11.5 \| 16.4 \| \| ADL limitations \| 24.9 \| 35.1 \| 33.5 \| \| Education<12 years \| 35.5 \| 71.4 \| 80.0 \| \| Annual family income<$9000 \| 18.7 \| 48.4 \| 55.5 \| \| Net assets<$25000 \| 18.7 \| 46.0 \| 52.0 \| \| No Health insurance \| 0.3 \| 0.9 \| 4.1 \| \| Medicare only \| 9.8 \| 32.6 \| 30.2 \| \| Medicare +Medicaid \| 5.0 \| 23.5 \| 37.3 \| \| Joint replacement history \| 8.0 \| 5.0 \| 6.0 \| | ***Two year arthritis-related JRT (Full cohort)***   \| **Variable** \| \| OR (95% CI) \| \| \| \| --- \| --- \| --- \| --- \| --- \| \| Age 80 and older \| \| 0.45 (0.29-0.70) \| \| \| \| Male* \| \| NS \| \| \| \| Arthritis at baseline** \| \| 7.77 (4.45 – 13.56) \| \| \| \| Other chronic conditions** \| \| NS \| \| \| \| Life threatening conditions** \| \| NS \| \| \| \| Physical limitations** \| \| NS \| \| \| \| ADL/IADL limitations** \| \| 2.48 (1.52-4.04) \| \| \| \| Education*** \| \| NS \| \| \| \| Income*** \| \| NS \| \| \| \| Wealth*** \| \| NS \| \| \| \| Medicare only*** \| \| 0.46 (0.22-0.95) \| \| \| \| **Black and Hispanic vs. Whites: adjusting variables** \| Full cohort N=6159 \| \| baseline arthritis N=1787 \| \| *Demographics \| 0.65 (0.35-1.18) \| \| 0.43 (0.23-0.78) \| \| *Demographic + **health needs \| 0.37 (0.20-0.71) \| \| 0.37 (0.19-0.72) \| \| *Demographic + **health needs + ***economic access \| 0.46 (0.22-0.98) \| \| 0.48 (0.21-1.08) \|   NS: only stated as not significant |
| George, Ruiz and Sloan, 2008 [20] | \| **Variable** \| THA  (N=131) \| No THA (N=257) \| \| --- \| --- \| --- \| \| Male \| 33.6 \| 30.7 \| \| Age 60-74 (youngest group) \| 40.5 \| 32.3 \| \| Age 85 and older \| 12.2 \| 20.6 \| \| Education<9 years \| 14.5 \| 22.2 \| \| Education≥13 years \| 42.0 \| 33.5 \| \| Stooping Difficulty/Not able \| 68.0 \| 50.9 \| \| Lifting Difficulty/Not able \| 35.9 \| 31.9 \| \| Walking Difficulty/Not able \| 61.1 \| 35.6 \| \| IADL limitations –Light housework \| 28.2 \| 26.1 \| \| IADL limitations –Heavy housework \| 62.6 \| 60.3 \| \| IADL limitations – Preparing meals \| 21.4 \| 22.2 \| \| IADL limitations – Shopping \| 32.1 \| 26.8 \| \| ADL limitations –Bathing \| 26.7 \| 23.0 \| \| ADL limitations -Getting in & out chair \| 32.1 \| 26.1 \| \| ADL limitations –Dressing \| 19.8 \| 14.0 \| \| ADL limitations –Walking \| 58.0 \| 45.4 \| \| ADL limitations –Use of toilet \| 16.8 \| 12.5 \| \| Income, $, mean (SD) \| 26500(191) \| 26470(215) \| \| Medicare + Private insurance \| 87.0 \| 79.0 \| \| Medicare +Medicaid \| 5.3 \| 16.3 \| \| Self reported health –Fair or poor \| 31.3 \| 30.7 \| \| BMI >30 kg/m2 \| 18.3 \| 20.6 \| \| Comorbidities –Parkinson’s disease \| 1.5 \| 1.9 \| \| Comorbidities – Alzheimer’s disease \| 1.5 \| 2.3 \| \| Comorbidities –Osteoporosis \| 26.7 \| 28.4 \| | ***Use of primary THA up to 3 years and 6 months after baseline interview***   \| **Variable** \| OR (95% CI) \| \| --- \| --- \| \| Age 85 and older \| 0.25 (0.12-0.50) \| \| Male \| NS \| \| Education \| NS \| \| Stooping \| 4.35 (2.24-8.42) \| \| Walking Limitations \| 2.63 (1.18-5.87) \| \| Lifting \| NS \| \| ADL/IADL limitations \| NS \| \| Race \| NS \| \| Married \| NS \| \| Income \| NS \| \| Medicare only \| NS \| \| Self-rated health \| NS \| \| Comorbid illnesses \| NS \| \| Time between baseline and follow-up interview \| NS \|   NS: only stated as not significant. |
| Hanchate et al., 2008 [21] | \| **Variable** \| White  N=14440 \| Black N=2586 \| Hispanic N=1413 \| \| --- \| --- \| --- \| --- \| \| Male \| 44 \| 62 \| 57 \| \| Age 47-64 (youngest group) \| 52 \| 58 \| 63 \| \| Arthritis \| 56 \| 61 \| 52 \| \| Hypertension/HBP \| 45 \| 63 \| 45 \| \| Diabetes \| 12 \| 23 \| 21 \| \| Cancer \| 12 \| 9 \| 6 \| \| Lung disease \| 9 \| 7 \| 6 \| \| Heart disease \| 21 \| 20 \| 14 \| \| Difficulty walking 1 block \| 9 \| 16 \| 11 \| \| Difficulty getting up from chair \| 33 \| 40 \| 37 \| \| Difficulty climbing 1 flight stairs \| 12 \| 20 \| 19 \| \| Stooping or crouching \| 38 \| 42 \| 38 \| \| Education<High school \| 18 \| 43 \| 59 \| \| Equiv. household income<10000 \| 13 \| 39 \| 46 \| \| Currently employed \| 35 \| 35 \| 39 \| \| Net assets<$5,000 (w/o house) \| 15 \| 52 \| 52 \| \| No Health insurance, age ≤64 \| 14 \| 17 \| 3 \| \| No health insurance, age ≥65 \| 0. \| 2 \| 2 \| \| Medicare only, age ≤64 \| 3 \| 6 \| 3 \| \| Medicare only, age ≥65 \| 42 \| 46 \| 44 \| \| Medicare +Medicaid, age≤64 \| 0.8 \| 3 \| 2 \| \| Medicare +Medicaid, age≥65 \| 3 \| 15 \| 28 \| \| Joint replacement history \| 0 \| 0 \| 0 \| | ***Receipt of Total Knee Arthroplasty over two years***   \| **Variable** \| OR (95% CI) Total sample (N=18439) \| OR (95%CI) Arthritis Subsample (N=10510) \| \| --- \| --- \| --- \| \| Black, female \| 0.94 (0.67-1.32) \| 0.94 (0.67-1.31) \| \| Hispanic, female \| 0.87 (0.54-1.43) \| 0.94 (0.57-1.54) \| \| White, male \| 0.88 (0.75-1.04) \| 1.01 (0.86-1.19) \| \| Black, male \| 0.56 (0.33-0.95) \| 0.65 (0.38-1.11) \| \| Hispanic, male \| 1.08 (0.65-1.81) \| 1.39 (0.83-2.33) \| \| Age 47-64 \| 0.72 (0.52-1.01) \| 0.80 (0.61- 1.06) \| \| Overweight or obese \| 2.61 (2.153.17) \| 2.39 (1.97-2.90) \| \| High blood pressure \| 1.11 (0.95-1.29) \| 1.02 (0.87-1.19) \| \| Diabetes \| 0.68 (0.56-0.85) \| 0.69 (0.56-0.86) \| \| Cancer \| 0.82 (0.65-1.03) \| 0.78 (0.62-0.99) \| \| Lung disease \| 0.85 (0.67-1.08) \| 0.79 (0.63-1.01) \| \| Heart disease \| 0.93 (0.78-1.10) \| 0.88 (0.74-1.05) \| \| Walking 1 block \| 1.53 (1.25-1.89) \| 1.55 (1.26-1.90) \| \| Getting up from chair \| 1.80 (1.51-2.14) \| 1.38 (1.17-1.64) \| \| Climbing 1 flight of stairs \| 1.41 (1.16-1.73) \| 1.40 (1.14-1.70) \| \| Stooping or crouching \| 3.05 (2.51-3.69) \| 2.21 (1.83-2.66) \| \| Health Insurance, age 65+, Medicare FFS only (reference) \| 1 \| 1 \| \| Medicare HMO/with Medicaid \| 1.28 (0.94-1.75) \| 1.28 (0.94-1.74) \| \| Medicare with private/DoD \| 1.18 (0.93-1.49) \| 1.18 (0.93-1.49) \| \| Health insurance, age <65, Private (reference) \| 1 \| 1 \| \| Uninsured \| 0.61 (0.40-0.92) \| 0.63 (0.41-0.94) \| \| Medicaid \| 1.53 (1.03-2.26) \| 1.45 (0.98-2.14) \| \| Household income <$20,000 \| 0.79(0.65-0.95) \| 0.77 (0.64-0.93) \| \| Household assets <$20,000 \| 0.86 (0.69-1.08) \| 0.63 (0.67-1.06) \| \| Employed \| 1.17 ((0.95-1.44) \| 1.28 (1.04-1.58) \| \| Education <High school \| 0.73 (0.58-0.91) \| 0.69 (0.55-0.87) \| \| GED or high school \| 1.02 (0.86-1.20) \| 0.98 (0.83-1.16) \| |
| Hawker et al., 2000 [30] Hawker et al., 2001 [31]; Hawker et al., 2002 [32];  Hawker et al., 2004 [33] | \| **Variable** \| Women (N=742) \| Men (N=248) \| \| --- \| --- \| --- \| \| No of self-reported conditions (SD) \| 1.38 (1.3) \| 1.45 (1.4) \| \| Self-reported cardiovascular disease \| 28.3 \| 35.5 \| \| Self-reported lung disease \| 16.7 \| 15.3 \| \| Spoke with physician about having JRT \| 32.5 \| 41.8 \| | ***Definite Willingness to have JRT***   \| **Variable** \| OR (95% CI) \| OR (95% CI) \| \| --- \| --- \| --- \| \| Age 65 and over \| 0.57(0.41 -0.79) \|  \| \| Age (per year increase) \|  \| 0.94(0.90-0.98) \| \| Had spoken with physician about JRT \| 2.93(1.78 -4.82) \| 3.19(1.63-6.23) \| \| Residence in a high rate of use area \| 1.30(0.78-2.18) \| 1.36(0.68-2.70) \| \| Male Sex \| 0.73(0.42-1.28) \| 0.47(0.19-1.13) \| \| Knows someone who has undergone JRT \| 1.76 (0.75-4.13) \|  \| \| WOMAC quantiles (reference is lowest) \| 1.007 (0.85-1.20) \|  \| \| Racial background \| NS \|  \| \| Income: $20001-40000  Income: ≤$20001 \|  \| 0.49(0.17-1.39)  0.36(0.11-1.15) \| \| Education level (reference post high school): High school only  Less than high school \|  \| 0.82(0.28-2.38)  1.19(0.50-2.86) \|   NS: only stated as not associated with outcome. Language spoken at home was analysed but results not reported. |
| Hawker et al., 2006 [26] | \| **Variable** \| Overall  (N=2128) \| Urban (N=970) \| Rural (N=1158) \| \| --- \| --- \| --- \| --- \| \| Age, mean (SD)) years \| 71 (9) \| 72 (10) \| 71 (9) \| \| Women \| 73.4 \| 74.7 \| 72.2 \| \| White \| 96.1 \| 92.3 \| 99.3 \| \| Living alone, independently \| 31.0 \| 39.0 \| 24.3 \| \| Education < high school \| 32.1 \| 28.6 \| 35.0 \| \| Income<$20,000/year \| 64.6 \| 64.8 \| 64.3 \| \| WOMAC score, mean (SD) \| 41.1 (20) \| 41.6 (20) \| 40.6 (19) \| \| Body Mass Index, mean (SD) \| 27.7 (5.4) \| 27.5 (5) \| 27.9 (5) \| \| One comorbid condition \| 29.9 \| 30.5 \| 29.4 \| \| ≥2 comorbid conditions \| 42.4 \| 41.8 \| 43.0 \| \| SF-36 General Health, mean(SD) \| 47.8 (22) \| 47.9 (23) \| 47.7 (22) \| \| Employed \| 4.1 \| 3.9 \| 4.3 \| \| Seen physician in the past year for hip/knee problems \| 48.1 \| 52.1 \| 44.8 \| \| Definitely/probably willing to consider surgery \| 20.0 \| 19.3 \| 20.6 \| | ***Predictors of time to TJA in individuals with no prior Total Joint Arthroplasty***   \| **Variable** \| Hazard Ratio (95% CI) \| \| --- \| --- \| \| Age quintile \|  \| \| ≤62 \| 1.00 (reference) \| \| 63-68 \| 1.57 (1.10-2.25) \| \| 69-74 \| 1.46 (1.01-2.10) \| \| 75-81 \| 1.51 (1.03-2.20) \| \| ≥82 \| 0.44 (0.22-0.88) \| \| WOMAC score -per 10-point increase \| 4.35 (2.24-8.42) \| \| SF-36 General Health subscale –per 10 point increase in score \| 1.14 (1.07-1.21) \| \| Willingness to consider TJA as a treatment option: \|  \| \| Unsure/unwilling \| 1.00 (reference) \| \| Willing \| 4.92 (3.73-6.44) \| \| Missing information \| 0.88 (0.57-1.34) \| |
| Ibrahim et al. 2002 [40]; Ibrahim et al., 2001 [39]; Ibrahim et al., 2002 [41] ; Lopez et al., [42] | \| **Variable** \| African American (N=262 \| Whites (N=334) \| \| --- \| --- \| --- \| \| Age mean years (SD) \| 65 (10) \| 66 (19) \| \| Male sex \| 100 \| 100 \| \| High school diploma or higher \| 57.4 \| 71.0 \| \| Employed (FT/PT/casual) \| 8 \| 15 \| \| Annual Household income<$10,000 \| 41.0 \| 20 \| \| Married \| 39 \| 56 \| \| WOMAC mean, 0-100 (SD) \| 46 (17) \| 45 (17) \| \| Kellgren-Lawrence 0-1 \| 48.9 \| 53.5 \| \| Kellgren-Lawrence 2-3 \| 51.1 \| 46.5 \| \| GDS score mean, 0-15 (SD) \| 4.5 (3) \| 5.0 (4) \| \| Charlson Comorbidity Index mean, 0-13(SD) \| 2.3 (2) \| 2.5 (2) \| | ***Willingness to consider joint replacement African-American vs White, adjusted for different sets of factors***   \| **Variable** \| OR (95% CI) \| \| --- \| --- \| \| Unadjusted \| 0.50(0.30 -0.84) \| \| Adjusted for core* variables \| 0.54(0.30 -0.96) \| \| Adjusted core* + Familiarity with TJA \| 0.53(0.30-0.96) \| \| Adjusted core* + Familiarity with TJA + Expectations of TJA \| 0.86(0.45-1.63) \|   * Age, level of education, annual income, radiologic severity of disease, WOMAC, geriatric depression score |
| Johnson et al., 2008 [61] | \| **Variable** \| New hip pain  GP-referred patients (N=52) \| \| --- \| --- \| \| Female sex \| 62 \| \| Age mean, years (SD) \| 64.6 (13.5) \| \| Referral dates \| June 2003-August 2004 \| | Authors state that information on medical history, current medications and the Oxford Hip Score (OHS) as well as hip radiographs used in the initial assessment of patients were analysed according to ‘a modified Kellgren-Lawrence criteria’ These data were then analysed to ‘demonstrate the OHS range and radiographic changes that were common to most patients who had been put on the waiting list for primary THA via the traditional out-patient route (and thus develop a the new fast track approach). Predictors were thus:  OHS: 34, and Radiological change: complete loss of joint space and or severe marginal osteophyte formation. |
| Judge et al., 2010 [26] | Original Sample was that of the Hospital Episode Statistics with TJR and English Longitudinal Study of Aging (ELSA), no information is provided on summary statistics of characteristics of those in need with and without TJR | ***Equity in Access ARR: Adjusted rate ratio***   \| **Variable** \| ARR TKA \| ARR THA \| \| --- \| --- \| --- \| \| Age 50-59 (reference) \| 1 \| 1 \| \| Age 60-69 \| 2.78 (2.69-2.87) \| 3.73 (3.59-3.88) \| \| Age 70-79 \| 2.43 (2.35-2.51) \| 4.25 (4.08-4.41) \| \| Age 80-84 \| 1.64 (1.57-1.71) \| 2.65 (2.53-2.78) \| \| Age≥85 \| 0.68 (0.65-0.72) \| 0.87 (0.82-0.93) \| \| Male Sex \| 1.08 (1.05-1.10) \| 1.31 (1.28-1.34) \| \| Peri-urban area (Town and fringe) \| 0.94 (0.91-0.98) \| 0.76 (0.73-0.79 \| \| Village/Isolated \| 1.16 (1.12-1.22 \| 0.92 (0.88-0.96) \| \| Deprivation Level 5 (most) to1 (least \| 0.31 (0 30  .33) \| 0.33 (0.31-0.34) \| \| No of hip operations/year/hospital trust (hip 570-1076 ref: ≤234; knee 503-803 ref: 30-204)) \| 1.13 (1.06- 1.20) \| 1.08 (1.02-1.15) \| \| Orthopaedic training centre \| 1 04 (0.99-1.09) \| 1.10 (1.05-1.16) \| \| Hip: 46-550 consultants per 100000pop,  ef: 4-30; Knee 47-448, ref:4-30 \| 0.80 (0.74-0.86) \| 0.92 (0.86-0.97) \| \| Hip: 3-14 Orthopedist per 100,000 pop, ref:1-2; Knee 3-11, ref: 1-2 \| 1.07 (1.02-1.04) \| 1.05 (0.99-1.11) \| \| Hip: 6-42 operating theatres/ 100,000 pop , ref 0-4; knee 1-4, ref:0-0.4 \| 1.11 (1.03-1.19) \| 1.11 (1.05-1.19) \| \| Distance to hospital (road travel times in mins) 46-226 (reference 2-13) \| 1.11 (1.05-1.17) \| NA \| |
| Juni et al., 2010 [36]; Juni et al., 2003 [37] | \| **Variable** \| Women (N=835) \| Men (N=467) \| \| --- \| --- \| --- \| \| Age mean years (SD) \| 62.8 (13) \| 61.8 (12) \| \| White ethnicity \| 98.4 \| 98.9 \| \| Manual occupation \| 73.0 \| 67.6 \| \| Married \| 60.9 \| 78.7 \| \| New Zealand Score mean (SD) \| 26.6 (19) \| 24.8 (20) \| \| Severe self reported comorbidity \| 5.9 \| 8.0 \| \| Unwilling to accept surgery \| 65.8 \| 59.7 \|   ***Hip pain study sample*** | ***Knee Pain: Willingness to undergo surgery if offered***   \| **Variable** \| OR (95% CI) \| \| --- \| --- \| \| New Zealand Score (p/10-point increase) \| 1.57 (1.47-1.66) \| \| Age (per 10-year increase) \| 0.71 (0.65 -0.77) \| \| Female Sex \| 0.60 (0.49- 0.74) \|   ***Hip Pain: Female to Male Sex ORs of access to health care***   \| **Dependent Variable** \| OR*  (N=1312) \| OR**  (N=1312) \| OR*  (N=746) \| \| --- \| --- \| --- \| --- \| \| Unwilling to undergo surgery if offered \| 1.48 (1.12-1.95) \| Not Applicable \|  \| \| Ever sought care from GP \| 0.78 (0.61-1.00) \|  \|  \| \| On drug therapy in previous 12 months \| 0.96 (0.74-1.24) \|  \| 0.96 (0.71-1.31) \| \| Ever referred to specialist \| 0.53 (0.40-0.70) \| 0.51 (0.38- 0.69) \| 0.52 (0.38-0.72) \| \| Consulted orthopaedic surgeon in past 12 months \| 0.55 (0.32-0.78) \| 0.51 (0.32-0.82) \| 0.56 (0.35-0.88) \| \| Awaiting hip replacement \| 0.41 (0.20-0.87) \| 0.53 (0.23-1.23) \| 0.41 (0.19-0.87) \|   * had sought GP; adjusted for age and severity (New Zealand score); **adjusted for age, severity, willingness and fitness for surgery (presence of selected comorbidities) |
| Lievense et al., 2007 [19] | \| **Variable** \| Patients with new hip pain  (N=224) \| \| --- \| --- \| \| Female sex \| 73.1 \| \| Age mean(SD), years \| 65.6 (10) \| \| BMI, mean(SD) \| 27.5 (5) \| \| Pain severity VAS (0-10), mean(SD) \| 6.1 (2) \| \| Pain ≥7 \| 42.4 \| | ***Factors determining THA within three and six years of first evaluation***   \| **Variable** \| OR THA 3 years  (N=193) \| OR THA 6 years  (N=163) \| \| --- \| --- \| --- \| \| Age≥60 years \| 9.9 (1.0-96.0) \| 3.5 (0.9-14.3) \| \| Morning stiffness \| 5.3 (1.5-18.2) \| 1.6 (0.6- 4.3) \| \| Worst pain location: Groin \| 1.6 (0.4-5.9) \| 2.8 (1.0- 8.1) \| \| Medial tight \| - \| 14.1 (0.4- 457) \| \| BMI≥30 kg/m2 \| 0.1 (0.02-0.6) \| 0.3 (0.1-1.0) \| \| Decreased active hip motion with: \|  \|  \| \| Extension \| 2.9 (0.8-10.1) \| 2.2 (0.8-6.4) \| \| Adduction \| - \| 2.1 (0.8-5.8) \| \| Painful passive internal rotation \| 3.0 (0.7-13.2) \| - \| \| Radiology: Kellgren/Lawrence score≥2 \| 7.0 (1.9-25.8) \| 8,6 (3.0-24.6) \| |
| Linsell et al., 2005 [52] | \| **Variable** \| Hip cases (N=212) \| Knee cases (N=612) \| \| --- \| --- \| --- \| \| Age group, years: 65-74 \| 63.7 \| 55.7 \| \| 75-84 \| 29.7 \| 36.6 \| \| 85+ \| 6.6 \| 7.7 \| \| Sex: Female \| 56.6 \| 55.2 \| \| Education: School \| 38.5 \| 35.1 \| \| Degree \| 15.5 \| 10.9 \| \| Professional \| 29.4 \| 23.8 \| \| BMI≥30: Male \| 9.4 \| 14.1 \| \| BMI≥30: Female \| 13.8 \| 20.5 \| \| Joints affected: Bilateral \| 21.9 \| 40.2 \| \| Lequesne functional index: Severe/Extreme \| 48.1 \| 63.6 \| \| Pain score: Moderate/Severe \| 57.1 \| 63.1 \| \| Other problems at least as bad as hip/knee \| 59.6 \| 48.8 \| \| Time since onset of joint problem  (median months) \| 24.0 \| 48.0 \| | ***Knee vs Hip Pain: Willingness to undergo TKA/THA if offered***   \| **Variable** \| OR (95% CI) \| \| --- \| --- \| \| Knee vs. Hip \| 1.12 (0.51-2.47) \| \| Age, Sex, Severity, Comorbidity, Bilaterality, Time of onset \| Not Stated \|   ***Knee vs Hip Pain: Knows someone with a TKA/THA***   \| **Variable** \| OR (95% CI) \| \| --- \| --- \| \| Knee vs. Hip \| 0.21 (0.13-0.35) \| \| Age, \| Not stated \| \| Sex \| Not stated \|   ***Knee vs Hip Pain: Believes TKA/THA is generally successful***   \| **Variable** \| OR (95% CI) \| \| --- \| --- \| \| Knee vs. Hip \| 0.58 (0.35-0.97) \| \| Age, Sex, Whether knows someone with hip/knee replacement \| Not stated \| |
| Momohara et al., 2007 [23] | \| **Variable** \| Patients with pain or tenderness in knee joint  (N=955) \| \| --- \| --- \| \| Female sex \| 86.3 \| \| Age median (IQR), years \| 58 (51-66) \| \| BMI, median(IQR) \| 21.1 (19-23) \| \| Onset age of RA, median (years) \| 48 (39-57) \| \| RA duration, median (years) \| 8 (3-14) \| \| Pain severity VAS (0-10), median(IQR) \| 4.2 (2-6) \| \| Physician VAS (0-10), median (IQR) \| 4.7 (2-6) \| \| Functional disability, J-HAQ, median (IQR) \| 1 (0.4-1.5) \| \| Disease Activity Score-28 joint, median (IQR) \| 4.63 (3.9-5.5) \| \| Rheumatoid Factor, median \| 67 (24-149) \| | ***Cox Proportional Hazard rate for TKA***   \| **Variable** \| HR (95% CI) \| \| --- \| --- \| \| Age (years) \| 0.98(0.96 -0.99) \| \| Functional disability J HAQ \| 1.59(1.20 -2.10) \| \| VAS-pain \| 1.23(1.13-1.34) \| \| Rheumatoid Factor \| 2.19(1.14-4.21) \| |
| Riddle, Kong and Jiranek, 2009 [78] | \| **Variable** \| Persons w/o KRT after 2 years (N=749) \| Persons w/ KRT after 2 years (N=29) \| \| --- \| --- \| --- \| \| Age, mean (SD) years \| 61.6 \| 66.6 \| \| Sex: Female \| 59.1 \| 79.3 \| \| Education: High school graduate or less \| 18.0 \| 34.5 \| \| BMI≥30 \| 45.5 \| 69.0 \| \| Currently seeing a doctor for arthritis \| 19.7 \| 44.8 \| \| Kellgren-Lawrence knee OA grade 4 for most involved side \| 27.8 \| 75.9 \| \| Time to completion of 400m walk test (10 s increments) \| 317 (66) \| 379 (125) \| \| PCS SF-12 score \| 46 (10) \| 38 (10) \| \| MCS SF-12 score \| 53 (9) \| 58 (7) \| | Study employed random forest analysis to derive predictors of TKA over two years instead of multivariate OR synthesis measures due to the very small numbers of cases. The study found that the Modified Kellgren-Lawrence Grade, PCS score from SF-12 and the 400 m walk time test score were the most influential predictors. |
| Schonberg et al., 2009 [46] | \| **Variable** \| Patients with severe hip/knee OA  (N=174) \| \| --- \| --- \| \| Female sex \| 76.4 \| \| Age median, years (range) \| 74 (65-96) \| \| Race: Non-Hispanic white \| 82.2 \| \| Non-Hispanic black \| 13.2 \| \| Other \| 4.6 \| \| Education: High school or less \| 45.1 \| \| At least some college \| 54.9 \| \| Income, US$25,000 or less \| 40.8 \| \| Income, more than US$25,000 \| 19.5 \| \| Marital status: Married \| 39.0 \| \| Not married \| 61.0 \| \| Lives alone \| 46.3 \| \| WOMAC, total score, mean (SD) \| 56.3 (13.2) \| | Study states that ‘Non-Hispanic whites were more likely to report that their primary care doctors recommended surgery than individuals from other races, although these results were not significant when income and education were adjusted for in a multivariable regression analysis’  Among those who saw a surgeon, ‘non-Hispanic whites were also more likely to report that their orthopaedists recommended surgery than other individuals of other races although these findings were also not significant when income and education were adjusted for in the model’ |
| Steel et al., 2008 [38] | \| **Variable** \| 1998  (N=10801) \| 2000 (N=10520) \| 2002 (N=10585) \| \| --- \| --- \| --- \| --- \| \| Age, 60-64 years \| 28.2 \| 28.3 \| 28.2 \| \| Age, 65-74 years \| 40.8 \| 41.1 \| 41.9 \| \| Age, 75 and older \| 30.9 \| 30.6 \| 29.9 \| \| Women \| 56.9 \| 57.3 \| 57.4 \| \| White \| 83.4 \| 83.0 \| 82.8 \| \| Attended college \| 34.7 \| 35.8 \| 37.3 \| \| Lowest third in wealth \| 29.4 \| 29.5 \| 30.2 \| \| Body Mass Index≥30 \| 19.7 \| 20.3 \| 23.0 \| | ***Receipt of TJR over two years***   \| **Variable** \| OR (95% CI) \| \| --- \| --- \| \| Age 60-64 \| 1 \| \| Age 65-74 \| 1.06 (0.64 -1.76) \| \| Over 74 \| 0.86 (0.48- 1.54) \| \| Female sex \| 0.97 (0.61-1.54) \| \| Ethnicity: Black \| 0.34 (0.17-0.66) \| \| Ethnicity: Other non-white \| 0.48 (0-11-2.03) \| \| No college education \| 0.65 (0.42-1.00) \| \| Lowest third wealth \| 0.97 (0.54-1.75) \| \| Middle third wealth \| 1.27 (0.76-2.04) \| \| BMI≥30 \| 1.32 (0.88-2.00) \| \| Currently in work \| 1.29 (0.74-2.25) \| \| Seen doctor ≥2 times in last 2 y \| 2.18 (0.52-9.15) \| \| Difficulty walking 1 block \| .37 (0.91-2.07) \| \| Married/Cohabiting \| 1.43 (0.87-2.34) \| \| Grandchild care \| 1.15 (0.73-1.80) \| \| Comorbidity \| 0.85 (0.57-1.28) \| |
| Suarez-Almazor et al., 2005 [44] | \| **Variable** \| White  (N=66) \| African American (N=66) \| Hispanic (N=66) \| \| --- \| --- \| --- \| --- \| \| Age, mean(SD) years \| 65.7 (7) \| 63.4 (6) \| 63.0 (5) \| \| Women \| 63 \| 62 \| 64 \| \| Education mean(SD), years \| 15.0 (2) \| 14.3 (3) \| 11.3 (3) \| \| Income mean(SD), X $1,000 \| 52 (23) \| 39 (23) \| 39 (25) \| \| Disease duration, mean(SD) years \| 5.9 (8) \| 4.7 (5) \| 4.6 (5) \| \| Overall health VAS, mean(SD) \| 76.2 (19) \| 68.6 (18) \| 75.1 (17) \| \| WOMAC mean(SD):Pain \| 43.4 (23) \| 53.3 (21) \| 37.5 (21) \| \| Stiffness \| 52.5 (26) \| 59.5 (25) \| 37.5 (23) \| \| Function \| 42.8 (23) \| 52.1 (21) \| 35.9 (22) \| \| SF-36 General Health, mean(SD): PCS \| 39.0 (10) \| 34.3 (10) \| 39.2 (9) \| \| MCS \| 55.2 (7) \| 51.2 (10) \| 53.2 (8) \| | ***Predictors of ‘Yes’ to Question 1: ‘Have you ever considered having knee replacement?’ and to Question 2: ‘If your knee arthritis became worse and your doctor recommended a knee replacement, would you consider having this surgery?’***   \| **Variable** \| Question 1  OR (N=198) \| Question 2  OR (N=198) \| \| --- \| --- \| --- \| \| Ethnicity: White \| 1.00 \| 1.00 \| \| African American \| 0.20 (0.07-0.57) \| 0.28 (0.05-1.50) \| \| Hispanic \| 0.46 (0.18-1.20) \| 0.15 (0.03-0.79) \| \| Age \| NS \| NS \| \| Female sex \| 2.70 (1.10-6.30) \| NS \| \| WOMAC pain \| 1.03 (1.00-1.05) \| NS \| \| Physician had recommended \| 39.2 (11.3-136.8) \| - \| \| Trust in physician (0-10) \| 1.40 (1.10-1.70) \| NS \| \| Perception of efficacy (1-5) \| 2.40 (1.70-5.50) \| 5.50 (2.80-10.70) \| \| Perception of risk (1-5) \| NS \| NS \| \| Relative/friend with TKA \| NS \| NS \| \| Education, years \| NS \| NS \|   NS: Only stated as not significant (i.e. p>0.05) |
| Yong et al., 2004 [48] | Original sample was that in the age/sex registers of Swindon and Sheffield Health Authorities, no information is provided on summary statistics of characteristics of those in need with and without TJR. Patients were aged 65 and older, by design. | ***Probability of undergoing TKA over 18 months***   \| Age \| Percent (95% CI) \| \| --- \| --- \| \| 65-74 \| 9 (4-14) \| \| 75-84 \| 7 (4-12) \| \| 85+ \| 3 (1- 9) \|   ***Probability of undergoing TKA over 18 months***   \| Sex \|  \| \| --- \| --- \| \| Male \| 7 (3-14) \| \| Female \| 6 (4- 9) \|   ***Probability of undergoing TKA over 18 months***   \| Deprivation \|  \| \| --- \| --- \| \| No \| 7 (4-11) \| \| Yes \| 6 (3-10) \| |
| Zeni et al., 2010 [18] | \|  \| \| \| \| --- \| --- \| --- \| \| **Variable** \| Had TKA (N=40) \| No TKA (N=80) \| \| Age mean years (SD) \| 63.3 (8) \| 57.6 (10) \| \| Male sex \| 46 \| 50 \| \| BMI (kg/m2) \| 7.0 \| 6.4 \| \| Affected Joint (Bilateral) \| 46 \| 43 \| \| Timed Up and Go test, seconds mean (SD) \| 9.5 (2.3) \| 8.5 (2.5) \| \| Stair Climbing Task, second mean (SD) \| 19.4 (11) \| 14.4 (7) \| \| Knee Outcome Score–ADL Subscale 0-100 \| 51.1 (19) \| 61.5 (22) \| \| Quadriceps strength Inv (N/BMI) \| 18.9 (9) \| 24.2 (12) \| \| Extension ROM degrees mean (SD) \| 3 (6) \| 0 (4) \| \| Flexion ROM degrees mean (SD) \| 124 (11) \| 125 (18) \| | ***Factors determining TKA within two years of first evaluation***   \| Variable \| OR \| \| --- \| --- \| \| Age>60 \| 1.13 \| \| Knee extension ROM (degrees)>1 \| 1.23 \| \| KOS –ADLS (%)>50 \| 0.96 \| \| TUG \| NS \| \| SCT \| NS \| \| Quadriceps strength \| NS \|   NS: stated as discarded in backward regression analysis |
