# Supplementary material for: Determinants of demand for total hip and knee arthroplasty: a systematic literature review
Source: BMC Health Serv Res. 2012 Jul 30;12:225. doi: 10.1186/1472-6963-12-225 (PMC3483199; doi:10.1186/1472-6963-12-225)
Supplement: Additional file 4 — Table S2. Results of Quantitative Studies. This table contains the detailed results presented by individual studies alongside the summary characteristics of the study sample. Readers may refer to this file if they want to learn about the statistical details of the individual study findings. [file 1472-6963-12-225-S4.docx]

| **Table i Characteristics of included studies** | | | | | |  |
| --- | --- | --- | --- | --- | --- | --- |
| **Study ID**  **Author/Year** | **Country/Region/Study date** | **Aim or objective of the study** | **Patient**  **Population** | **Study**  **Type/setting** | **Study size** | **Measure of Need** |
| Allepuz 2008 [62] | Spain, Catalonia, Andalusia, Aragon Canary Islands.  2001- 2006 | Psychometrics of **surgeons**’ prioritization instrument for surgery. | Patients on the waiting list for hip and knee arthroplasty. | Observational Multicentre | 10 centres  944 patients  16 surgeons | Placement on waiting list |
| Birk and Henriksen, 2006 [54] | Denmark, Eastern, Roskilde County, 1999-2000 and 2003 | Reasons for patients to accept/decline re-referral to a distant hospital with shorter waiting time. | Patients on a waiting list for hip and knee replacement at the single public hospital in the study area which performs hip and knee replacements. | Cross-sectional (retrospective 3-year after the response) | 144 patients | Placement on a waiting list for surgery |
| Birrell et al., 2003 [22] | UK  1994- 1997 | Incidence and predictors of waiting list placement for THA | Consecutive patients attending GP practices with a new episode of hip pain. GPs had professional interest in rheumatology. | Longitudinal; Multicentre  Follow up: 3 years (median) | 195 patients | Pain in the bathing trunk area, arising from the hip joint structure |
| Borkhoff et al., 2008[55]; Borkhoff et al., 2009 [56] | Canada,  Toronto  2003-2005 | **Physicians’** recommendation about TKA to two clinically- identical, standardised moderate OA patients differing by sex. | Surgeons from opinion survey on TKA and GPs with open practices using the Canadian Medical Directory. | Cross sectional | 38 GPs  33 surgeons | Chronic knee pain having exhausted all non-operative treatment options |
| Boutron et al., 2008 [60] | France  2004- 2005 | Factors in **GPs**’ opinion that their patients will need prosthetic replacement within 1 year of consultation. | Patients > 45 years, seeking treatment for knee/hip OA with radiographic evidence, no previous surgery (excl. hip and knee OA cases). | Cross-sectional national survey | 1471 GPs  4121 patients  (Knee: 2540, Hip: 1581) | Arthritis related doctor visit and radiographic |
| Card, Dobkin and Maestas, 2008 [47] | US, California, Florida and New York  1992-2003 | Impact of Medicare eligibility on healthcare use. | People between the ages of 60 and 70 from Hospital discharge records. | Cross sectional | 453,000 hip and knee replacement admissions | None -implicitly matched for |
| Conner-Spady, 2008 [27]; Conner-Spady, 2007 [53] | Canada  Saskatchewan  Nov 2006 | Predictors of patients’ willingness to consider changing to a surgeon with a shorter waiting time for TKA/THA. | On waiting list/HRT/ KRT 3–12 months before, ≥18 years, in Surgical Patient Registry. Random sample, by group and joint strata. | Observational cross-sectional study | 468 waiting list patients  541 patients with prior surgery | Placement on waiting list |
| Cross, 2000 [79] | Australia  1994-1995 | WTP for JRT for OA in patients who had had the procedure. Convergent and Construct validity study. | OA patients who had had primary THA/TKA at three Sydney hospitals, covered privately, by Medicare (public), or Veterans Affairs. | Observational study | 109 THA pts.  129 TKA pts. | Patient after surgery |
| De Coster et al., 2007 [65] | Canada | Development of prioritisation tool for referrals by **primary care providers** | Orthopaedic surgeons and primary care doctors | Psychometric (prioritisation tool development) | 6 surgeons; 25 primary care providers | Not applicable |
| Derret, et al., 2002[63]; Derret et al., 2003 [64] | New Zealand | Whether prioritisation scoring was associated with patients’ access to surgery in practice | Consecutive patients assessed for hip or knee joint replacement surgery at a single centre | Observational study | 137 | CPAC (priority score)=40 (out of maximum score of 100) |
| Dosanjh, 2009 [51] | US  California  2005 | Patients’ decision-making process to undergo THA. | Patients either scheduled for or with completed THA. | Qualitative | 18 pts (5 post-surgery pts) | Placement on waiting list |
| Dunlop, et al., 2003 [29] | US  1993-1995 | To compare JRT use by black, Hispanics, & whites after 2 years, and access factors. | Older adults in the Community; AHEAD (a national probability sample) | Longitudinal national cohort | 6159 | Self-report of arthritis related visit to the doctor |
| Figaro, Russo and Allegrante, 2004 [43] | US  New York  Date not stated | Contribution of older urban African-Americans’ views on arthritis and surgery to observed disparities | Age> 50 years, with medical insurance, Black/African-American, Harlem residents, with pain/stiffness in knee(s) & walking difficulties. | Qualitative study | 94 patients | Pain/stiffness in knee(s) & walking difficult/slow in last 6 months |
| George, et al., 2008 [17] | US 1992-2003 | Effect of THA on physical functioning at 1 year. | Stratified random sample of OA Medicare beneficiaries, ≥65 years, without prior THA | Longitudinal national MCBS | THA: 131, No THA: 257 | ICD-9-CM: OA diagnosis |
| Gooberman-Hill et al., 2010 [49]; Sansom et al., 2010 [50] | UK, city not stated  2006 | Decision making within orthopaedic consultations focused on TJR | Patients seeing participating clinicians about hip/knee replacement, identified from clinic lists, from three hospital sites in a single city. | Qualitative: audio record of consultations in-depth interviews | 4 surgeons, 2 Extended Scope Practitioners  26 patients | Referral to specialist |
| Hanchate, et al., 2008 [18] | US 1994-2004 | To estimate national TKA rates and economic factors on racial and ethnic disparities in TKA use. | White, Black and Hispanic individuals born before 1942 and their spouses/partners, without history of TKA/THA | Longitudinal nationally, HRS | 18439 | Self-report of arthritis or rheumatism |
| Hawker, et al., 2000 [30]; Hawker, et at.,2001 [31]; Hawker et al.,2002 [32]; Hawker et al., 2004 [33]; | Canada,  Ontario  1999 | To determine unmet need differences between men and women and willingness to undergo surgery among patients in need. | Age≥55 yrs., general pop.; WOMAC >39, clinical, radiographic OA, no stroke with paralysis or major mental/neurological disease | Cross-sectional | Men: 15,819, Women: 21,337;Potential surgery Men:122; Women: 323 | WOMAC≥39 |
| Hawker et al., 2006 [26] | Canada,  Ontario  1999 and 1999-2003 | To prospectively examine the predictors of time to receipt of a first TJA, including willingness to operate. | General population cohort, Age≥55 yrs., WOMAC >39, clinical, radiographic OA, no stroke with paralysis or major mental/neurological disease | Longitudinal.  Follow up: 5 years | 2,128 | WOMAC≥39 |
| Ibrahim et al., 2002 [40],[41]; Ibrahim et al., 2001 [39], Lopez et al., [42] | US  1997-2000 | Causes of racial and ethnic disparities in THA and TKA in the US Veterans Affairs (VA) system | African American and white males in primary care at a Cleveland VA Medical Center, ≥50 years with hip and knee pain | Cross-sectional study | 596 patients (44% AA) | Lequesne score ≥5 (moderate to severe pain) for ≥6 months |
| Johnson et al., 2008 [61] | England, Surrey,  August 2002 and December 2005 | To identify clinical predictors of being put on waiting list for primary THA. | Patients referred to a hip specialist orthopaedic surgeon by GPs in the catchment area of a district general hospital. | Prospective case series | 50 ‘design’ &  52 ‘evaluation’ patients | Referral to orthopaedic clinic with hip pain problem |
| Judge, et al., 2010 [28] | England 2002-2003 | Geographical, socio-demographic THA/TKA access factors | Age≥50 yrs, general pop; New Zealand score≥48 (out of 80) | Cross-sectional ELSA & HES | 76,690 age-sex-wards | New Zealand score 48 (of 80), 43 or 53 |
| Juni et al., 2010 [36]; Juni et al., 2003 [37] | England 1998-1999  Somerset and Avon | Hip/knee patient gender differences in GP consultation, drug therapy receipt, referral to and consultation with specialist, and THA. | Age≥35 years with hip/knee pain on most days for ≥1 months over the previous 12 months, selected from 40 general practices in the Southwest of England | Cross-sectional SASH | 1,302 | New Zealand score 43 (moderately severe) and 55 (severe) |
| Karlson, et al., 1997 [35] | US  1993 -1994 | Sex differences in preferences for the timing of elective TJR in patients with moderately severe hip/knee OA | OA patients aged 60 or older, visiting the primary care doctor included in arthritis register; | Qualitative | Men: 12, Women: 18  Hip only: 7;Knee only: 15; Hip & Knee: 8 | Moderately severe OA by medical record & radiographic report evaluation |
| Lievense et al., 2007 [19] | The Netherlands,  Rotterdam  1996-2002 | Individual patient incidence of THA or severe symptoms and its predictors | Consecutive patients≥50 years of age presenting to their GP with hip pain persisting for 1 month-2 years, for whom GP requested a radiograph at one of two hospitals. | Prospective Longitudinal cohort  Follow-up at 3 & 6 years (postal survey) | 224 patients | WOMAC≤32 (mild), 32.1-64 (moderate), and >64 (severe) |
| Linsell et al. 2005 [52] | England, Oxfordshire  April 2002 | Differences in primary care management explaining higher rates of joint replacement with hip than knee pain | A random sample of Oxfordshire residents, aged 65 years and above, from the Oxfordshire Health Authority register. | Cross-sectional postal survey | 3341 respondents | Lequesne 8-13 (severe), and 14-24 (extremely severe) |
| McHugh and Luker, 2009 [24] | England, Northwest, 2007 | To elucidate some of the factors that influence the decision to have a TJR | Hip and knee OA, recruited from a specialist orthopaedic centre, newly referred by their GP to orthopaedic consultant | Qualitative  Purposive sample | 27 patients  (Knee: 10,  Hip: 17) | Referrals to specialist |
| Momohara et al., 2007 [23] | Japan, Tokyo, 2000 -2005 | To identify the risk factors for TKA in a cohort of RA patients followed for 5 years | Patients fulfilling the ACR criteria for RA, with pain or tenderness in their knee joints and without a previous TKA. | Prospective (time to TKA) | 955 patients | Pain or tenderness in knee joints |
| Quintana et al.,2006 [59]; Quintana et al., 2000 [57] ; Escobar et al., 2003 [58] | Spain  1999- 2000  2003- 2004 | Factors in **specialists**’ categorization of patients as appropriate for TKA/THA; relation of appropriateness to outcomes | Two groups of specialists, one for THA and one for THK  Consecutive pts scheduled to undergo THA. | Prospective observational cohort study  Follow-up 6 months | 584 THA patients  601 TKA patients | Not applicable |
| Riddle, Kong and Jiranek,  2009 [78] | US; Baltimore, Columbus, Pittsburgh and Pawtucket; year not stated | Estimate incidence and identify predictors of knee arthroplasty over two years in a cohort of symptomatic knee OA. | Symptomatic OA in one/both knees, recruited via mailings to clinical populations, adverts in local newspapers, visits to community centres and web. | Prospective observational cohort study (multicentre) | 778 patients | Frequent knee symptoms last year & OARSI atlas grade 1-3 (radiologic) |
| Sanders, Donovan and Dieppe, 2003 [34] | UK  1992-1994  1998-1999 | Patients’ perceptions of need for TJR, and barriers to healthcare for severe joint problem | Individuals with moderate to severe (NZ≥43) self-reported pain and disability, told by doctor that had hip/knee ‘OA’. | Qualitative, In-depth interviews | 46 patients Interview: Hip: 6, Knee: 21, Both:10 | New Zealand 43-55 (moderate to severe) |
| Schonberg, Marcantonio and Hamel, 2008 [46] | US, Boston, Massachusetts  2001-2006 | Whether older patients with severe OA report discussing TJA as a treatment option and its association with receipt of TJA. | Patients≥65 years in database with reports of radiographs from five sites, suggestive of severe OA; at least moderate pain, stiffness, activity limitations and functional impairment | Longitudinal observational cohort  Follow-up 12 months | 174 | >1 WOMAC activity limitation, functional impairment for ≥6 months, w/ medication |
| Steel et al., 2008 [38] | US  1998, 2000, 2002 | To determine rates of KRT, HRT among patients in need | US, age≥60, mobility limitations & severe pain, arthritis treatment, no TJR contraindication. | Longitudinal HRS  Follow-up two years | Men: 4400  Women: 1183 | Reported doctor diagnosis of OA |
| Suarez-Almazor et al., 2005 [44]; Suarez-Almazor et al., 2010 [45] | US, Houston, Texas.  2001-2002 | To assess the preferences and beliefs of patients with knee OA from diverse ethnic backgrounds in relation to TKA | Patients at one institution with ICD-9M code 715.90, knee OA diagnosis, white/African American/Hispanic, age≥55 years, no prior TKA, Spanish/ English proficient. | Cross sectional | White:66  African American: 66  Hispanic: 66 | Physician diagnosis of OA |
| Toye et al., 2006 [25] | UK; Not stated | To explore patients’ meanings of knee OA and TKA | Patients listed for TKA at one specialist orthopaedic hospital with < average pain & function. | Qualitative; interviews | 18 (12 men) | WOMAC Pain<52 &  Function<53 |
| Yong et al., 2004 [48] | England, Wiltshire and Sheffield | To quantify the effect of rurality and socio-economic disadvantage on need for knee replacement and the use of surgery. | Random sample of persons ≥65 years from two HA registers; BMI<30, without Parkinson’s, angina/heart, severe bronchitis/asthma disease, stroke/heart attack. | Baseline cross-sectional mail survey & Longitudinal tracing, Follow up 18 months | 574 | Lequesne Index  Need of specialist opinion: 14  Alternative:11 |
| Zeni 2010 [18] | US | Clinical predictors of decision to undergo TKA in OA patients. | End-stage knee OA with knee pain during daily activities & radiographic OA, from one surgeon. | Longitudinal observational | 120 | Knee pain; Kellgren-Lawrence≥3, > 1 compartment |

HA: Health Authority; ELSA: English Longitudinal Survey of Aging; HES: Hospital Episode Statistics; AHEAD: Asset and Health Dynamics Among the Oldest Old; OARSI: Osteoarthritis Research Society Intl.; MCBS: Medicare Current Beneficiary Survey; SASH: Somerset and Avon Survey of Health
