# Supplementary material for: Determinants of demand for total hip and knee arthroplasty: a systematic literature review
Source: BMC Health Serv Res. 2012 Jul 30;12:225. doi: 10.1186/1472-6963-12-225 (PMC3483199; doi:10.1186/1472-6963-12-225)
Supplement: Additional file 5 — Table S3. Disease Specific and Generic Health-Related Quality of Life Outcome Tools). This file presents a summary table describing the health related quality of life tools used by the studies reviewed. This information may help readers to interpret the detailed results in Additional file 4. [file 1472-6963-12-225-S5.docx]

| **Table iii Disease specific and Generic Health Related Quality of Life tools** | | | |
| --- | --- | --- | --- |
| **Tool** | | **Joint** | **Description** |
| Delaware  Osteo-arthritis profile | Quadriceps strength | Knee | Volitional force produced during unilateral isometric knee extension [15]. |
|  | Knee Outcome Survey-Activities of Daily Living (KOS-ADLS) | Knee | Subsection of KOS, a questionnaire about individual’s perception of ability to perform activities of daily living) [15]. |
|  | Timed Up and Go test (TUG) | Knee | A timed test in which the subjects rise from a chair, walk three meters, turn around and return to a seated position in the chair [15]. |
|  | Stair Climbing Task (SCT) | Knee | A times test in which the subjects begin at the bottom of a flight of twelve stairs, ascend the steps on the investigator’s command, turn around and descend the stairs [15] |
| WOMAC | | Knee and Hip | A self-reported measure of Pain, Mobility and Functioning involving 24 questions, 5 for pain, 2, for stiffness and 17 on physical function items. The responses are scored on a best to worst scale, so that lower scores represent less pain, less stiffness or better physical function. A total index score is commonly produced and transformed to a 0-100 scale [105]. |
| New Zealand score | | Knee and Hip | A index measure of need for surgery, constructed on the basis of self reported indicators of Pain (degree and occurrence; 40%), Functional activity (20%), Other factors (Multiple joint disease, Ability to work, give care, live independently; 20%) , and, information from clinical examination on Movement and Deformity (20%) [106]. |
| Lequesne Index | | Knee | A questionnaire comprising 10 questions., 5 of which relate to pain or discomfort, 1 question on maximum distance walked, and 4 questions on activities of daily living. Responses to the questionnaire are scored on a 0-24 scale, with lower scores representing less functional limitations [107]. Scores of 1-7, 8-13, and 14-24 define mild-moderate, severe and extremely severe states. |
| Oxford Hip Score | | Hip | A tool developed for self completion by patients undergoing THA consisting of 12 questions relating to pain and functional disability of the hip, experienced over the past 4 weeks, each with five response categories scored from 1 to 5. The total score is made up by the sum of the scores for individual items. Scores range from 12 to 60, with higher values representing worse pain and function [108]. |
| Euro Qol-5 Dimension (EQ-5D) | | Generic | A tool used to value health states on the basis of pain, anxiety/depression, mobility, self care and usual activities, with three severity levels, None, Some or Extreme (bed-ridden for mobility). The dimensions are combined into a single index using weights reflecting relative preference opinions developed from a survey of the general population. The tool is commonly used in analysis of resource allocation decisions across clinical areas [109]. |
| Medical Outcomes Study Short-Form 36-item (SF-36) | | Generic | A self completion questionnaire consisting of 36 items across eight components; physical function (10 items); role limitation due to physical problems (4 items); bodily pain (2 items); general health (5 items); role limitation due to emotional problems (3 items); social function (2 items); mental health (5 items); energy/vitality (4 items).It is commonly used to provide an eight-item profile of scores across the range of health domains, and physical and mental summary scores combining the first four and last four components are also used [110]. |
